# Live-cell imaging of human liver fibrosis using hepatic micro-organoids

**DOI:** 10.1172/jci.insight.187099

**Published:** 2024-12-10

**Authors:** Yuan Guan, Zhuoqing Fang, Angelina Hu, Sarah Roberts, Meiyue Wang, Wenlong Ren, Patrik K. Johansson, Sarah C. Heilshorn, Annika Enejder, Gary Peltz

**Affiliations:** 1 Department of Anesthesia, Pain and Perioperative Medicine, Stanford University School of Medicine, Stanford, California, USA.; 2 Department of Materials Science and Engineering, Stanford University, Stanford, California, USA.

**Keywords:** Hepatology, Fibrosis

## Abstract

Due to the limitations of available in vitro systems and animal models, we lack a detailed understanding of the pathogenetic mechanisms of and have minimal treatment options for liver fibrosis. Therefore, we engineered a live-cell imaging system that assessed fibrosis in a human multilineage hepatic organoid in a microwell (i.e., microHOs). Transcriptomic analysis revealed that TGFB converted mesenchymal cells in microHOs into myofibroblast-like cells resembling those in fibrotic human liver tissue. When pro-fibrotic intracellular signaling pathways were examined, the antifibrotic effect of receptor-specific tyrosine kinase inhibitors was limited to the fibrosis induced by the corresponding growth factor, which indicates their antifibrotic efficacy would be limited to fibrotic diseases solely mediated by that growth factor. Based upon transcriptomic and transcription factor activation analyses in microHOs, glycogen synthase kinase 3β and p38 MAPK inhibitors were identified as potential new broad-spectrum therapies for liver fibrosis. Other new therapies could subsequently be identified using the microHO system.

## Abstract 2

microHOs provide a novel multilineage, microwell-based hepatic organoid platform that can be used for investigating the pathogenesis of liver fibrosis, and for the discovery and characterization of the pharmacodynamic effect of anti-fibrotic drugs.

## Introduction

Liver fibrosis is a pathological condition caused by the accumulation of extracellular matrix (ECM) that develops in response to chronic liver injury (1). It results from an interaction between parenchymal and nonparenchymal liver cells and involves endogenous and liver-infiltrating immune cells (2–4). The key nonparenchymal cell is the myofibroblast, which is generated when a fibrogenic stimulus induced by liver injury causes hepatic stellate cells (HSCs) to transdifferentiate into myofibroblasts that produce fibril-forming collagens and other ECM components (4–8). Before organoid methodology was available, in vitro models often did not have the spectrum of cell types that mediate fibrogenesis, which limited our ability to explore fibrogenic mechanisms. Commonly used rodent models require an injury-inducing agent (carbon tetrachloride, bile duct ligation, or dietary modulation) and are very time-consuming and expensive to run, and any conclusions drawn from them are limited by concerns about their fidelity with the processes mediating the commonly occurring forms of human liver fibrosis (9). However, the pathogenesis of liver fibrosis can best be studied using a multilineage human hepatic organoid (HO) that has the cellular components and the geometry of hepatic lobules, which enables the effect that biochemical signals have on fibrosis to be quantitatively characterized. We previously developed an in vitro model system that differentiates human induced pluripotent stem cells (iPSCs) into a multilineage HO that has hepatocytes, cholangiocytes, HSCs, and cells of other lineages (fibroblasts, macrophages, and endothelial cells); other structures found in the liver lobule are formed in these HOs (10, 11). HOs engineered to express a causative mutation for autosomal recessive polycystic kidney disease (ARPKD) developed an extensive fibrosis (11) characterized by a marked increase in thick collagen fiber formation, which resembled that in liver tissue obtained from patients with ARPKD. The myofibroblasts in ARPKD organoids resembled those in the livers of patients with virally induced cirrhosis or advanced non-alcoholic steatohepatitis (NASH) (11). Here, we engineered a live-cell imaging system for identification of the collagen-producing cells in HOs that was adapted for use in a microwell-based platform. This system was used to characterize the signals driving liver fibrosis at the whole-organoid, single-cell, and molecular levels. Moreover, when the interaction between the intracellular signaling pathways activated by 2 pro-fibrotic factors were characterized, 2 potentially new approaches for treating liver fibrosis were identified.

## Results

### A live-cell imaging system for hepatic fibrosis.

**Figure 1:**
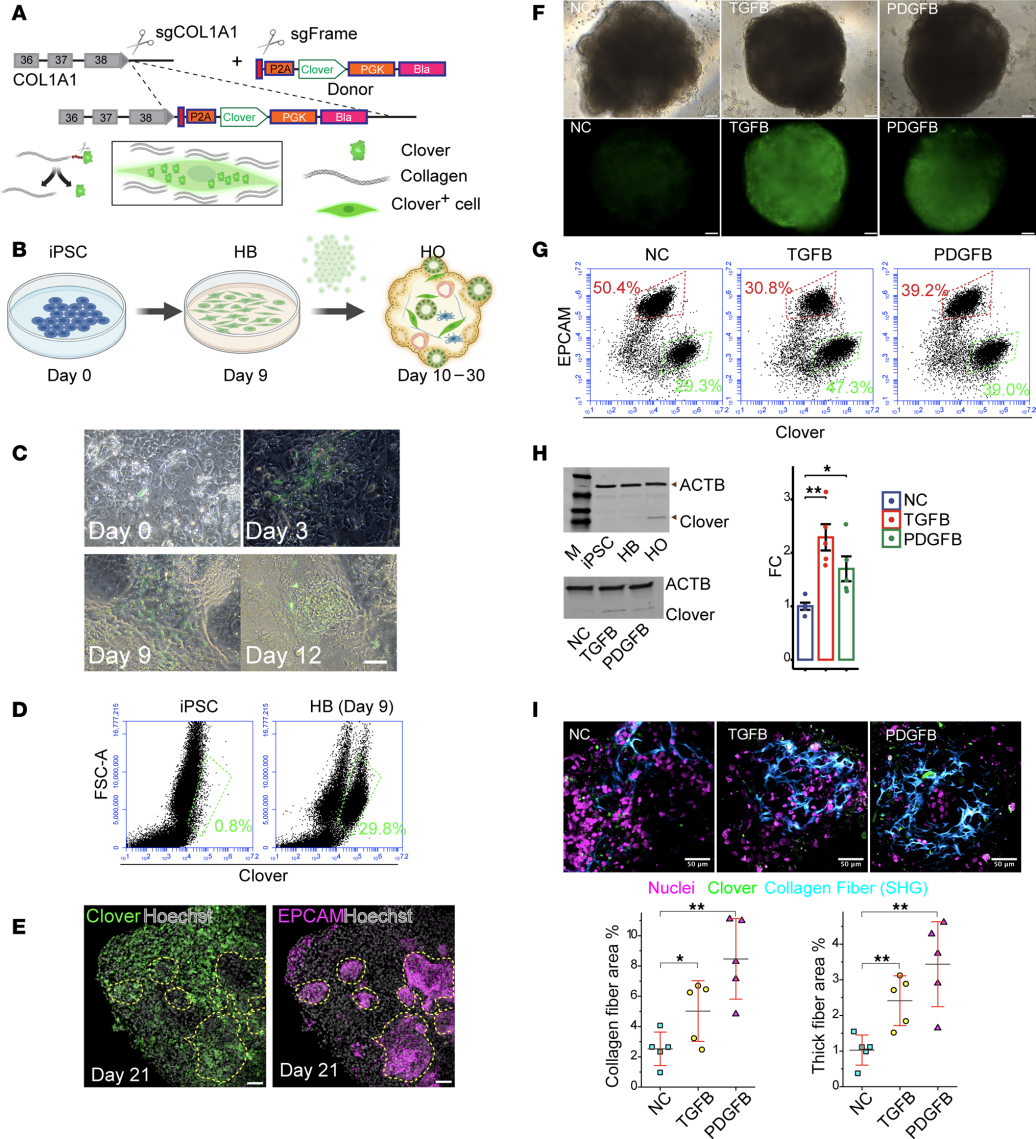
Generation of the *COL1A1*-P2A-Clover iPSC line used to monitor hepatic fibrosis. (**A**) *Top*: CRISPaint system used to insert P2A-Clover at the 3′ end of *COL1A1* in iPSCs. After blasticidin selection, cells were cloned to generate COL1A1-P2A-Clover iPSCs. *Bottom*: Self-cleaving P2A peptide enables *COL1A1*-expressing cell labeling with fluorescent intracellular protein (Clover). (**B**) Collagen-producing cells in an HO produced from *COL1A1*-P2A-Clover iPSCs labeled as in **A**. (**C**) Clover^+^ cells increase between days 3 and 12. (**D**) The Clover^+^ cell population is absent in iPSCs (day 0) but appears in (day 9) hepatoblasts in differentiating *COL1A1*-P2A-Clover HO cultures. (**E**) Location of antibody-stained fluorescent Clover^+^ or EPCAM^+^ cells in day 20 *COL1A1*-P2A-Clover organoids. Yellow dashed circles indicate areas with EPCAM^+^ (i.e., hepatocytes or cholangiocytes) cells; Clover^+^ cells are absent from those areas but present elsewhere in the HO. (**F**) Day 21 *COL1A1*-P2A-Clover HOs treated on day 13 with no addition (NC), 50 ng/mL TGFB, or 50 ng/mL PDGFB. Both growth factors induced a marked increase in COL1A1^+^ cells. (**G**) Clover^+^ cells are increased in day 21 *COL1A1*-P2A-Clover HOs after TGFB or PDGFB exposure and distinct from EPCAM^+^ (hepatocytes or cholangiocytes) cells in HOs. (**H**) *Left*: Clover protein expression in differentiating HO cultures (iPSC, day 9 hepatoblasts [HB]) and in day 21 control (NC), PDGFB-treated, or TGFB-treated HOs. Clover and β-actin (ACTB) proteins are indicated. *Right*: Fold-change in normalized Clover protein expression (relative to control HOs). Each data point is the average of 5 experimental repeats. (**I**) SHG analysis of collagen fibers in human HOs. *Top:* Cross-sectional view of collagen fibers within day 21 control organoids and organoids treated with TGFB or PDGFB on day 13. Control organoids (left) have isolated regions with relatively thin collagen fibers (blue). TGFB- or PDGFB-treated organoids form a network of thick collagen fibers extending throughout the organoid. Collagen-producing cells (green). *Bottom:* Quantitative comparison of collagen fiber area in SHG images for control, TGFB-treated, or PDGFB-treated hepatic organoids (*n* = 5/measurement; day 21). Increase in total collagen abundance in organoids after TGFB (*P < 0.05) or PDGFB exposure (**P < 0.01, Welch’s *t* test) and in abundance of thick collagen fibers (i.e., >3 μm diameter) in TGFB- and PDGFB-treated hepatic organoids. Scale bars, 50 mm (**C** and **E**); 50 μm (**F** and **I**).

Collagen is a triple helical protein that is composed of 2 COL1A1 (α1) chains and 1 COL1A2 (α2) chain, and increased collagen synthesis by activated myofibroblasts is a major contributor to liver fibrosis (12). To characterize fibrogenic mechanisms, we developed a live-cell imaging system, which enabled collagen-producing cells in HOs to be identified and quantitatively analyzed. A CRISPR-assisted insertion tagging system (CRISPaint) (13) was used to insert a Clover expression cassette (*COL1A1*-P2A-Clover) at the COOH terminus of the endogenous *COL1A1* gene in a control iPSC line. Since the insert has a self-cleaving P2A peptide, *COL1A1* mRNA–expressing cells in HOs produced from *COL1A1*-P2A-Clover iPSCs were labeled with a fluorescent intracellular protein (Figure 1, A and B). The Clover^+^ cell population increased in HO cultures at the hepatoblast stage (day 9) (Figure 1, C and D). Immunostaining and flow cytometry revealed that Clover^+^ cells were markedly increased in day 21 HOs that were treated with TGFB or PDGFB on day 13, coexpressed PDGFRβ and COL1A1, were present throughout the organoid, and were distinct from hepatocytes or cholangiocytes (Figure 1, E–G, and Supplemental Figure 1; supplemental material available online with this article; https://doi.org/10.1172/jci.insight.187099DS1). The flow cytometry results verified that Clover fluorescence was cell based (Figure 1, D and G). The pro-fibrotic effect of TGFB and PDGFB was consistent with the temporal pattern of expression of mRNAs encoding their receptors (*PDGFRA*, *PDGFRB*, *TGFBR1*, *TGFBR2*, *TGFBR3*, *BMPR1B*) and intracellular signaling proteins (STATs, SMADs), which were expressed at the hepatoblast stage (day 9) (i.e., before exposure to PDGFB or TGFB on day 13) and in mature HOs (Supplemental Figure 2). Clover protein was expressed at an extremely low level in iPSCs. Its expression was slightly increased in hepatoblasts, but it was more abundantly expressed in control, TGFB-treated, and PDGFB-treated mature HOs (Figure 1H).

The spatial distribution and morphology of the collagen fibers in HOs were examined using second harmonic generation (SHG) microscopy (14, 15), which has previously been used to quantitatively analyze liver fibrosis in human patients (16, 17) and to monitor fibrosis in ARPKD HOs (11). Of importance, SHG measures thick collagen fiber formation. This is a complex biochemical pathway that requires the coordinated activity of multiple liver enzymes that include proteases and other posttranslational processing enzymes that act on collagen, and subsequently, the activity of lysyl hydroxylases and lysyl oxidases to generate thick collagen fibers (18). SHG images revealed that control HOs had sporadic networks of cross-linked collagen fibers surrounding the cells in some isolated regions, while the TGFB- or PDGFB-treated organoids had more extensive networks of thick collagen fibers throughout the entire HO. A quantitative comparison indicated that TGFB (*P* < 0.05) or PDGFB (*P* < 0.01) exposure induced statistically significant increases in total collagen abundance and in the abundance of thick collagen fibers (i.e., >3 μm diameter) formed in HOs (*P* < 0.01) (Figure 1I). The SHG and immunostaining results indicate that PDGFB or TGFB increases the numbers of collagen-synthesizing cells and of thick collagen fibers formed in HOs.

### A high-content screening system for serially monitoring the effect of biochemical signals on liver fibrosis.

**Figure 2:**
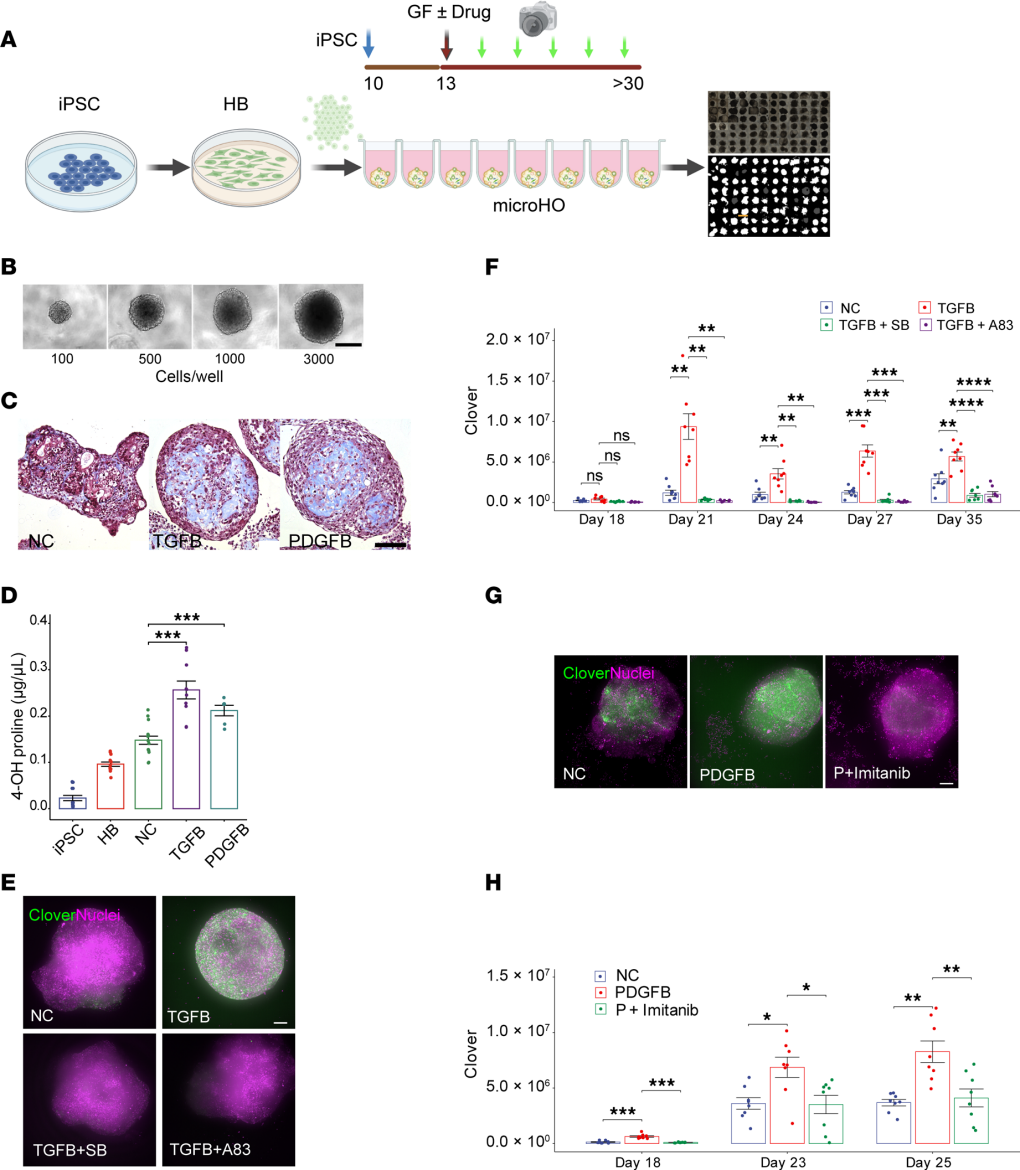
Establishment of a high-content imaging platform for antifibrotic drug screening. (**A**) High-content screening (HCS) platform for microHO analysis. COL1A1-P2A iPSCs are differentiated into hepatoblasts (HBs), and 10,000 HBs/microwell are placed on day 10. Growth factors (GF) and/or drugs are added on day 13, and cultures are differentiated into HOs. Culture fluorescence is serially assessed to quantify COL1A1-producing cells in the microHO. (**B**) Brightfield images on day 12 reveal microHOs have a uniform shape, and their size depends upon the number of input cells. Scale bar, 100 μm. (**C**) Trichrome staining reveals a marked increase in collagen (blue) in PDGFB- or TGFB-treated microHOs relative to control (NC) microHOs. Scale bar, 50 μm. (**D**) The 4-hydroxyproline (4OH-Proline) concentration was measured in differentiating HO cultures (iPSC, day 9 HB) and in day 21 control (NC), PDGFB-treated, or TGFB-treated HOs. The bar graph shows the mean ± SEM of measurements performed on a total of 15 HOs, which were generated in 2 separate experiments. There was an increase in 4OH-Proline in the PDGFB- or TGFB-treated day 21 HOs (vs. NC, *t* test). (**E**) Maximum intensity projection (MIP) images of *Z*-stack sections obtained from the indicated type of microHOs on day 21. Scale bar, 100 μm. Clover expression is green, and nuclei stained with Hoechst 33342 are purple. (**F**) TGFB-induced fibrosis in microHOs is blocked by TGFBR1 inhibitors. *COL1A1*-P2A-Clover HOs were formed by adding 10,000 HBs to each microwell. Then, either nothing (NC) or 50 ng/mL TGFB ± 10 μM TGFBR1 inhibitor (SB431542 [SB] or A83-01 [A83]) was added to each microwell on day 13. COL1A1^+^ cells within a microHO were serially measured on days 18 through 21. Each dot represents a measurement made on 1 microHO, and 8 microHOs per treatment were assessed per condition. A 2-way ANOVA indicates that drug treatment and time are 2 variables that have a significant interaction on the fluorescence measurements (*P* = 1.66 × 10^–15^) (Supplemental Table 9). (**G**) Representative MIP images obtained from the indicated type of microHO on day 21. Scale bar, 100 μm. (**H**) The PDGFB-induced increase in COL1A1^+^ cells is blocked by a PDGFRβ inhibitor. On day 13, 50 ng/mL PDGFB (P) or 50 ng/mL PDGFB with 10 μM imatinib was added to each microHO. *P < 0.05; **P < 0.01; ***P < 0.001; ****P < 0.0001.

Culture conditions were modified to enable HOs to reproducibly grow to a standard size in microwells, which we refer to as “microHOs” (Figure 2, A and B). Trichrome staining verified that TGFB or PDGFB induced a large increase in collagen in microHOs (Figure 2C). The TGFB effect on thick collagen fiber formation was validated by 4-hydroxyproline (4OH-Pro) measurements: 4OH-Pro was absent in iPSCs; it increased in day 9 hepatoblasts and in day 21 control HOs but was significantly increased after PDGFB or TGFB exposure (*P* < 0.001 vs. control) (Figure 2D). HCS was performed using a confocal fluorescence microplate imager with an automated program that we developed for analyzing multiple *Z*-stacked confocal images. The TGFB concentration used to induce fibrosis was determined to be 50 ng/mL (Supplemental Figure 3). TGFB addition on day 13 induced a sustained increase in COL1A1^+^ cells in microHOs that was blocked by coadministration of TGFBR1 tyrosine kinase inhibitors: 10 μM SB431542 (19, 20) or A83-01 (21) (Figure 2, E and F). PDGFB addition on day 13 also increased the number of COL1A1^+^ cells in microHOs, which was blocked by addition of a PDGFRβ tyrosine kinase inhibitor: 10 μM imatinib (22) (Figure 2, G and H).

The microHO system was also used to examine the effect of other agents that had been directly or indirectly implicated in the pathogenesis liver fibrosis: 5 cytokines — IL-4 (23), IL-6 (24), IL-13, IL-33 (25, 26), and TNF-α (27) — and 3 growth factors — insulin-like growth factor (IGF-1) (28), VEGF (29), and CCL3 (30). None of these agents induced fibrosis in microHOs (Supplemental Figure 4). Analysis of the temporal pattern of receptor expression in differentiating HO cultures using previously obtained transcriptomic data (11) revealed that the mRNAs encoding the IGF-1 (*IGF1R*), IL-13 (*IL13RA1*), and VEGF (*VEGFR-1* [*FLT1*]; *VEGFR-2* [*KDR*]) receptors were expressed in day 9 hepatoblasts and in mature (day 21) organoids. In contrast, the mRNAs encoding the receptors for IL-33 (*IL1RL1* or *ST2*), IL-4 (*IL4R*), or the common γ chain that is used for intracellular signaling by multiple cytokine receptors (*IL2RG*) were not expressed in day 9 hepatoblasts (Supplemental Figure 5A). Of note, the mRNAs encoding most of these receptors were abundantly expressed in mature HOs (Supplemental Figure 5B). Hence, the lack of a profibrotic effect of IGF-1 and VEGF was not due to the absence of receptor expression when microHOs were exposed to these agents. However, the lack of a profibrotic effect of IL-4 and IL-13 could result from the absence of functional receptors on day 9, which is just before the time of exposure to those cytokines. Since hedgehog signaling pathway activation increases *COL1A1* mRNA expression in myofibroblasts (31) and promotes myofibroblast accumulation and liver fibrosis (32), we examined whether a hedgehog pathway activator would induce fibrosis in microHOs. Smoothened (SMO) is a 7-transmembrane protein (33, 34) that accumulates in the primary cilium and transduces a hedgehog pathway signal intracellularly. However, addition of an SMO agonist, which activates hedgehog signaling (35), did not induce fibrosis in microHO cultures (Supplemental Figure 4).

### The Clover^+^ population represents mesenchymal cells and myofibroblasts.

**Figure 3:**
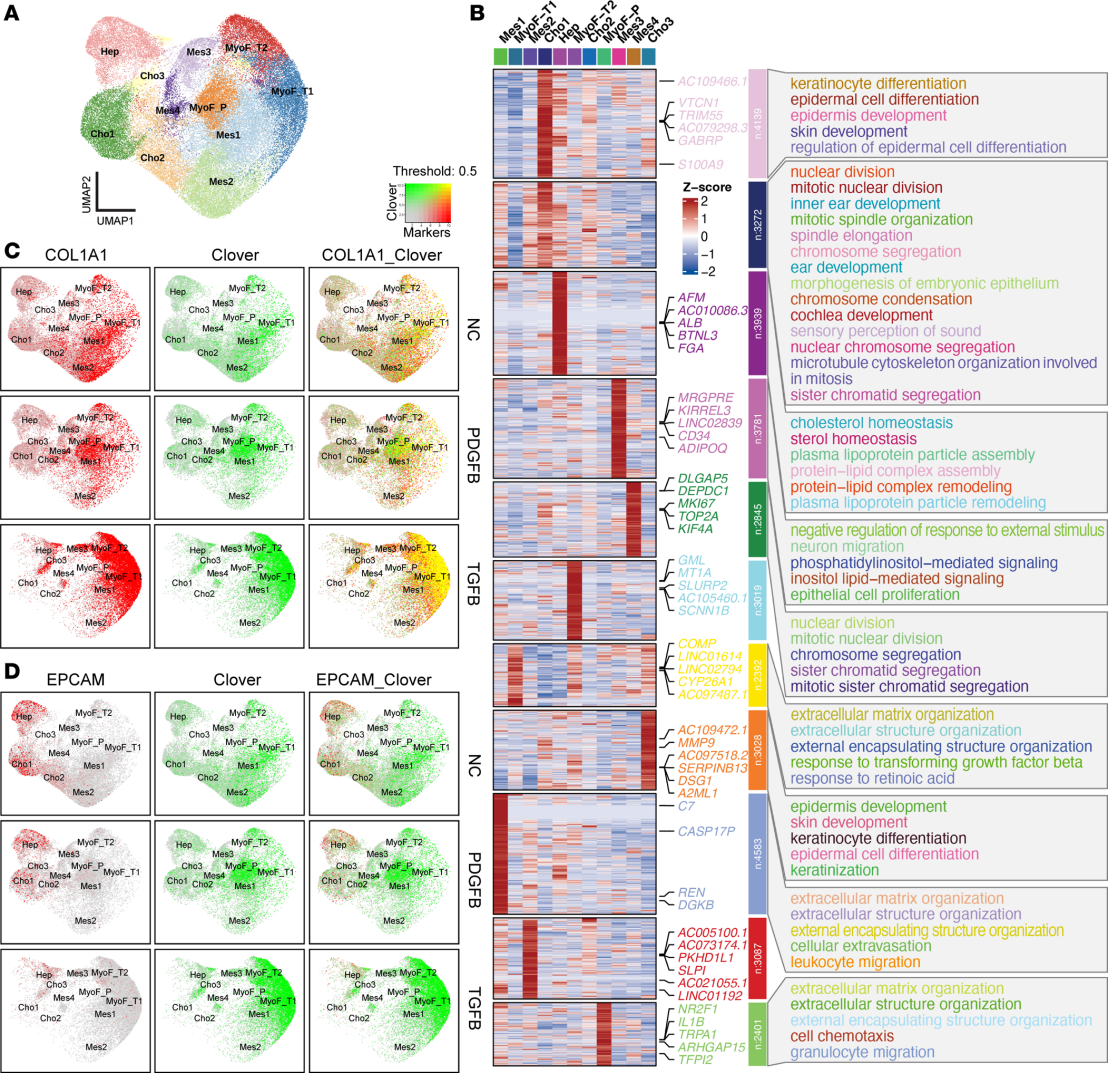
scRNA-Seq profiles microHOs and reveals the Clover^+^ population represents mesenchymal cells. (**A**) A combined sample uniform manifold approximation and projection (UMAP) plot shows the 11 cell clusters identified in day 21 control (NC), PDGFB-treated (P), and TGFB-treated (T) microHO cultures. (**B**) Heatmaps show the differentially expressed genes and the annotated GO pathways identified for each cluster. (**C** and **D**) These feature plots show the level of expression of *COL1A1* and *Clover* mRNAs (**C**) or *EPCAM* and *Clover* mRNAs (**D**) in the UMAP plot shown in **A**. As shown in the color threshold diagram, dot colors represent level of mRNA expression. *COL1A1* and *Clover* mRNAs have an overlapping expression pattern; they are predominantly expressed in myofibroblasts and in mesenchymal cells. In contrast, *EPCAM* mRNA is expressed in the hepatocyte and cholangiocyte clusters, and its expression does not overlap with that of *Clover* mRNA.

To characterize the pro-fibrotic effects of PDGFB and TGFB, single-cell RNA-Seq (scRNA-Seq) data were generated from day 21 control (22,211 cells), TGFB-treated (17,554 cells), or PDGFB-treated (18,013 cells) microHOs. We identified 11 cell clusters by analysis of this scRNA-Seq dataset (Figure 3A and Supplemental Table 3). We analyzed the differentially expressed genes and the Gene Ontology (GO) biological process annotations for each cluster to identify the cell type each cluster represented (Figure 3B, Supplemental Figure 6, and Supplemental Table 4). Based upon expression of canonical mRNAs and by the level of concordance between their transcriptomes with the cell types in human liver, the microHOs had cholangiocyte (Cho1-3), hepatocyte (Hep), mesenchymal cell (Mes1-4), and myofibroblast (MyoF_T1-2, MyoF_P) clusters. Also, *COL1A1* and *Clover* mRNAs had an identical pattern of expression in the microHOs: both mRNAs were expressed in myofibroblasts and mesenchymal cells. In contrast, an epithelial mRNA (*EPCAM*) that is a hepatoblast and cholangiocyte marker was not expressed in the *Clover* mRNA^+^ cells (Figure 3, C and D). Many mRNAs found in cholangiocytes (*KRT19*, *KRT7*, *CFTR*) or in hepatocytes and cholangiocytes (*HNF4A*, *AFP*, *CDH1*, *ALDHA1*, *APOB*, *FGB*) were also not expressed in the *Clover* mRNA^+^ cells in the microHOs (Supplemental Figure 7). Myofibroblast marker mRNA (*ACTA2*, *PDGFRA*, *FAP*) expression was very low in control microHOs but was predominantly expressed in the myofibroblast (MyoF_T1/2, MyoF_P) clusters in PDGFB- or TGFB-treated microHOs (Supplemental Figure 8). Consistent with the profile of *FAP* mRNA expression in microHOs (Supplemental Figure 8), FAP is absent in most tissues under basal conditions but is highly expressed on activated myofibroblasts (36, 37). FAP is a well-established marker for activated fibroblasts (38), and its serine protease activity promotes liver fibrosis by activating HSCs and promoting macrophage infiltration after liver injury (39).

### Changes in mesenchymal and epithelial cell clusters in fibrotic microHOs.

**Figure 4:**
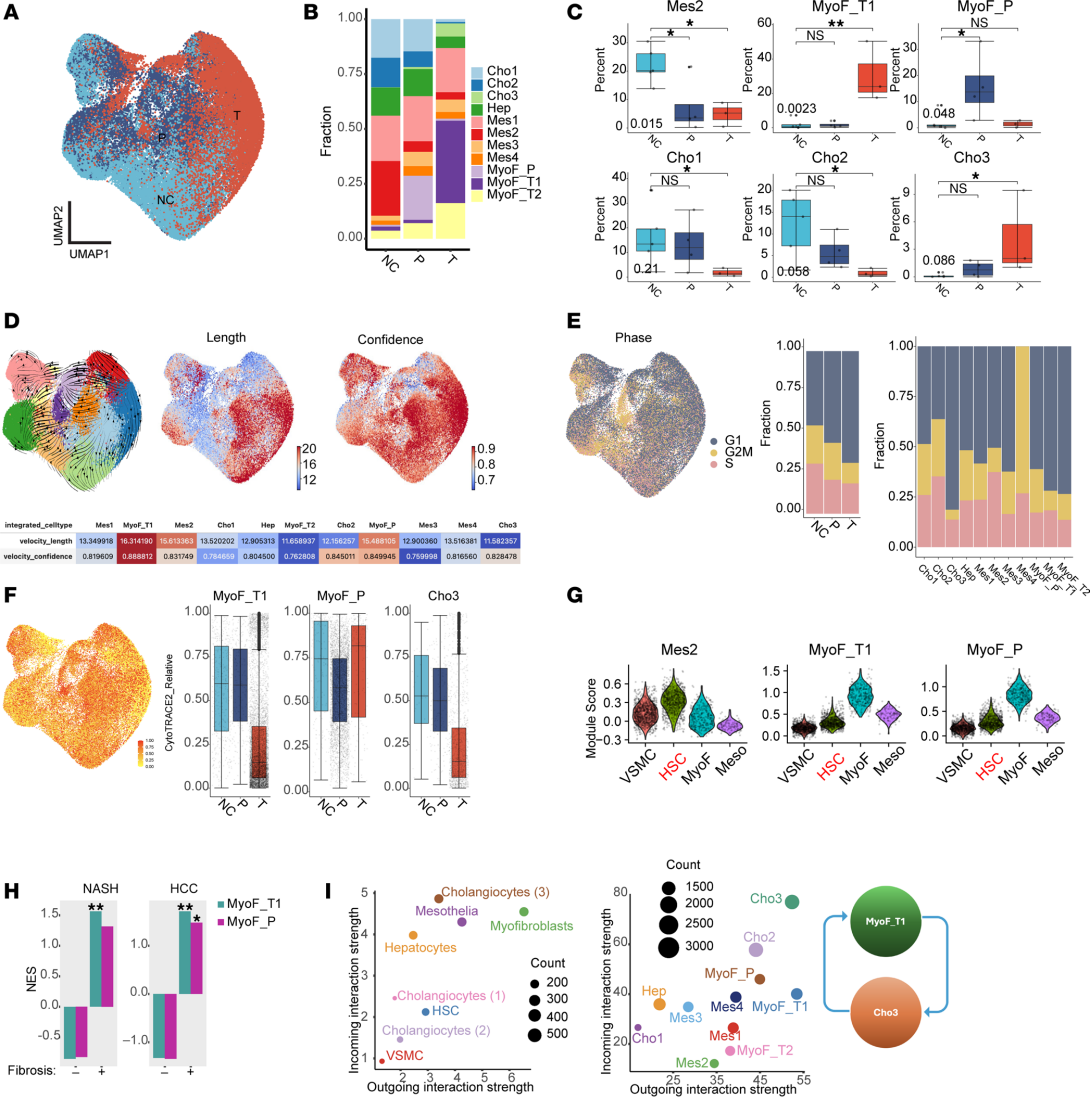
PDGFB- and TGFB-induced cellular composition changes in mesenchymal and epithelial cell clusters in fibrotic microHOs. (**A**) Combined UMAP for scRNA-Seq from cells in day 21 control (NC), PDGFB-treated (P), or TGFB-treated (T) microHO cultures. (**B**) Cell percentages for each of the 11 clusters in the types of microHOs. (**C**) Percentage change of each indicated cell cluster in NC, PDGFB-treated, and TGFB-treated microHOs. (**D**) RNA velocity results plotted on a UMAP. *Left*: stream embedding; *Middle*: velocity length indicates differentiation rate; *Right*: confidence, determined by correlation of velocity determined for neighboring clusters in UMAP. *Bottom*: velocity summary for all clusters. (**E**) Cell cycle phase on the UMAP. Percentage of cells in each phase of the cell cycle for NC, PDGFB-treated, and TGFB-treated microHOs or for all 11 clusters. (**F**) CytoTRACE score plotted on the UMAP or as box plots for MyoF_T1, MyoF_P, and Cho3 cells in NC, PDGFB-treated, and TGFB-treated microHOs. (**G**) Among cell types in human liver, MyoF_T1 and MyoF_P transcriptomes are most similar to myofibroblasts, while Mes2 is most similar to HSCs. Results when Mes2, MyoF_T1, and MyoF_P gene signatures were compared with transcriptomes of the following cell types in normal and cirrhotic (90) human liver: vascular smooth muscle (VSMC), HSC, MyoF, and mesothelium. MyoF_T1 and MyoF_P module scores are most similar to MyoF; Mes2 has greatest similarity with HSCs. MyoF module scores for MyoF_T1 and MyoF_P are 3-fold higher (*P* < 1 × 10^–10^) than that of HSCs; HSC gene signature module score for Mes2 is 4.8-fold (*P* < 1 × 10^–10^) higher than for MyoF. (**H**) Normalized enrichment score (NES) of GSEA examining the association of MyoF_T1 and MyoF_P gene signatures with liver tissue from nonfibrotic or fibrotic NASH liver and with resected HCC tissue (nonfibrotic or fibrotic). The MyoF_T signature was strongly associated with fibrotic NASH liver; both signatures were strongly associated with fibrotic HCC but not with nonfibrotic NASH or HCC tissue. (**I**) Cell type interactions (CellChat). Axes in left and middle represent total outgoing or incoming information associated with each cell type from all CellChat signaling pathways or only the “COLLAGEN pathway.” *Right*: Hypothesis generated by CellChat analysis. The highest level of intercellular communication occurs between MyoF_T1 and cholangiocytes. *P < 0.05; **P < 0.01.

To systematically examine the changes in the cell types in PDGFB- or TGFB-treated microHOs (vs. NC), 1-way ANOVA and sccomp, a statistical model using a constrained β-binomial distribution (40), were used to statistically analyze the changes in cell type proportions. The cells in PDGFB- or TGFB-treated microHOs were reproducibly different from those in control microHOs (Figure 4, A and B, and Supplemental Figure 9). One myofibroblast cluster (MyoF_T1) was far more abundant in TGFB-treated microHOs than in control or PDGFB-treated microHOs (37.5% of total vs. <2%), while another myofibroblast cluster (MyoF_P) was more abundant in PDGFB-treated microHOs than in control or TGFB-treated microHOs (20% of total vs. <2%). The abundance of a mesenchymal cluster (Mes2) was markedly decreased in TGFB- or PDGFB-treated microHOs (25% vs. <5%) (Figure 4C and Supplemental Table 3). A 1-way ANOVA comparing the different types of cells in each of 5 independent biological replicates analyzed by scRNA-Seq indicated that MyoF_T1 (*P* < 0.015) and MyoF_P (*P* < 0.05) abundances were significantly increased, while Mes2 abundance was decreased (*P* = 0.004) in TGFB- or PDGFB-treated microHOs. The sccomp analysis indicated that the Cho1-2 clusters were significantly decreased, while Cho3 was significantly increased in TGFB-treated microHOs (Supplemental Table 5 and Supplemental Figure 10).

To better understand the TGFB effect on the mesenchymal and epithelial cell clusters, their differentiation state and other properties were characterized using 3 computational methods for analysis of scRNA-Seq data. (a) RNA velocity (41), which assessed the differentiation state of the cell clusters, indicated that MyoF_T1 was the most highly differentiated cluster (Figure 4D). (b) Cell cycle analysis indicated that TGFB-treated microHOs had the highest percentage of G1 phase cells, which indicates that the cells were less proliferative. At the cluster level, MyoF_T1 and Cho3 had the highest percentage of cells in G1 and the lowest percentage of G2/M and S phase cells, which indicates that those clusters were less proliferative (Figure 4E). (c) Consistent with the RNA velocity results, CytoTRACE2 (42) indicated that MyoF_T1 and MyoF_P were the most differentiated of the mesenchymal cell clusters, whereas Cho3 was the most differentiated epithelial cluster (Figure 4F). These results indicate that MyoF_T1 cells are the most differentiated of the mesenchymal cell populations in fibrotic microHOs, and they have a low level of proliferation after they exit the cell cycle (Figure 4E).

Transcriptomic comparisons with various cell types in control and cirrhotic human liver were performed to further characterize the 3 clusters whose abundance was significantly altered by TGFB or PDGFB exposure. The results revealed that MyoF_T1 and MyoF_P clusters were most like those of myofibroblasts, whereas Mes2 was most similar to HSCs in human liver (Figure 4G, Supplemental Figure 11, and Supplemental Tables 6 and 7). As described in Supplemental Data 1, gene set enrichment analysis (GSEA) (43) revealed that the gene expression signature of MyoF_T1 in microHOs resembled that found in fibrotic human liver tissue caused by NASH or hepatocellular carcinoma (HCC) (Figure 4H, Supplemental Figure 12, and Supplemental Table 8). While the MyoF_P gene signature was positively associated with NASH- or HCC-induced liver fibrotic tissue, those GSEA associations did not achieve statistical significance. Also, the pattern of expression of 9 mesenchyme- and myofibroblast-specific mRNAs in mesenchymal cells and myofibroblasts in human liver was retained in Mes2 and MyoF_T1 cells in microHOs (Supplemental Figure 13). Taken together, the transcriptomic results indicate that TGFB or PDGFB exposure converts a population of HSC-like cells into myofibroblast-like cells in microHOs. Since nonparenchymal cells (i.e., the mesenchymal cells in microHOs) and epithelial cell interactions play a key role in liver fibrosis, CellChat (44) was used to examine cell cluster interactions in fibrotic microHOs and in cirrhotic human liver. It was noteworthy that the myofibroblasts in cirrhotic liver (or MyoF_T1 in microHOs) and cholangiocytes in cirrhotic liver (or Cho3 in microHOs) had the strongest incoming and outgoing interactions (Figure 4I), which indicates that these cells and pathways could be targeted for pharmacologic intervention. To address the origin of the mesenchymal cells present in microHOs, we examined scRNA-Seq data obtained from iPSC, day 9 hepatoblast, and mature HO cultures (Supplemental Figure 14). Since a distinct subset of cells in the hepatoblast cultures express mRNAs that are mesenchymal cell markers, the mesenchymal cells develop from iPSCs at an early differentiation stage.

### Identification of drug targets through analysis of TF activation.

**Figure 5:**
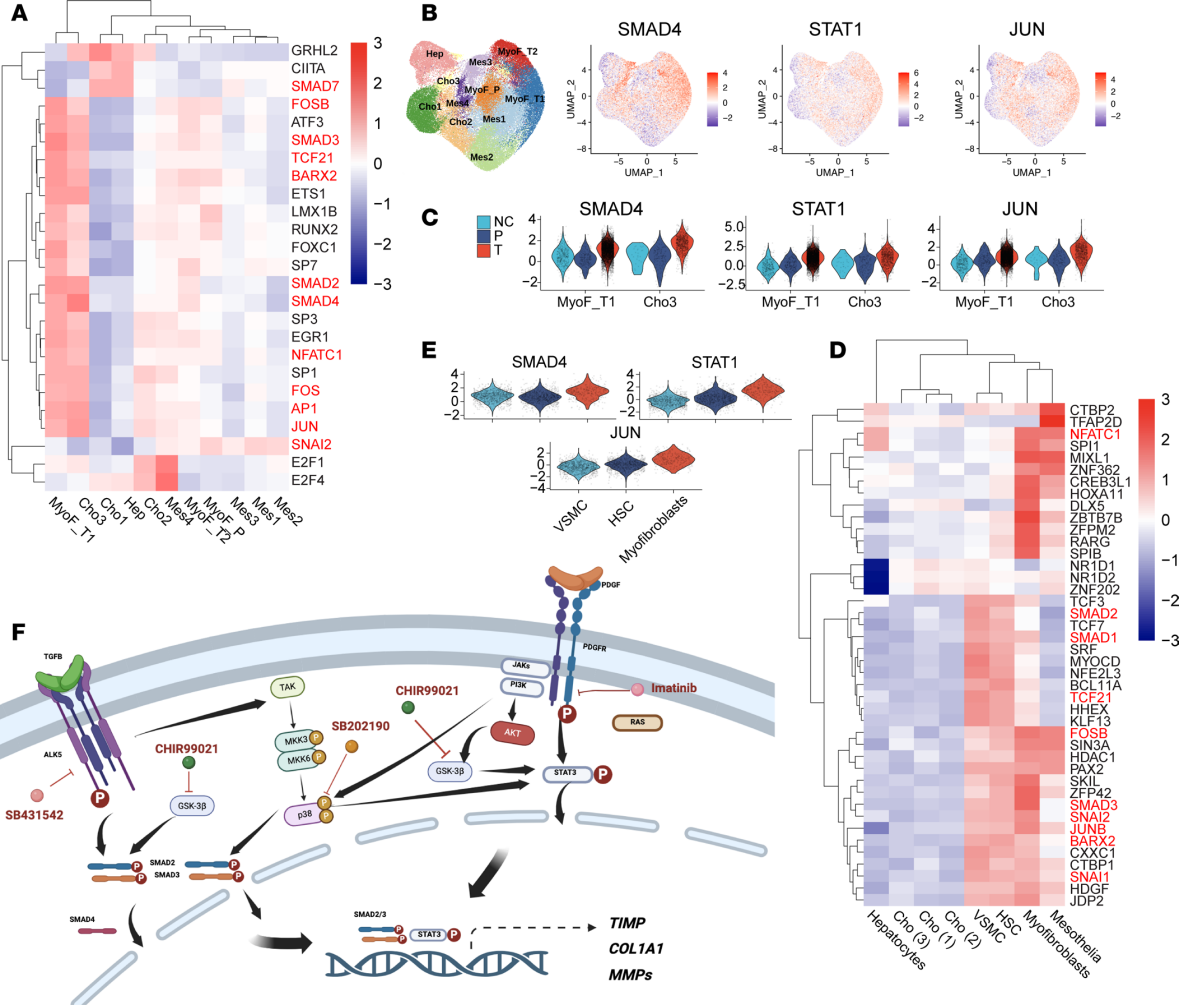
The TFs activated in fibrotic microHOs and in fibrotic human liver. (**A**) A heatmap shows the mean activity of the 25 TFs that were identified by decoupleR as being the most activated in fibrotic microHOs. The scaled mean activity for each TF in a cell cluster is indicated by the color of the square. Of note, MyoF_T1 and Cho3 had the highest level of activation of these TFs. (**B**) SMAD4, STAT1, and JUN activity was plotted on the UMAP, and the cluster regions are indicated on the UMAP shown on the left. The scaled mean activity for each TF is indicated by dot color. The MyoF_T1 cluster had the highest level of activity of these TFs. (**C**) Violin plots show the TGFB-induced increased TF activity in the MyoF_T1 and Cho3 clusters. (**D**) A heatmap shows the mean activity of the 42 TFs that were identified by decoupleR as being the most activated in fibrotic human liver. The scaled mean activity for each TF in a cell type is indicated by the color of the square. Myofibroblasts have the most activated TFs, and there is overlap with the TFs activated in MyoF1 in fibrotic microHOs (highlighted in red). (**E**) Violin plots show that myofibroblasts have an increased level of SMAD4, STAT1, and JUN activity versus that of VSMCs or HSCs. (**F**) Potential targets for antifibrotic drugs within the intracellular signaling pathways that are activated by TGFB or PDGFB. Pro-fibrotic agents activate the SMAD and STAT pathways, which can also activate the p38 MAPK and GSK3β pathways as shown in the diagram. Several potential targets for antifibrotic agents within SMAD, STAT, and interacting (p38 MAPK and GSK3β) pathways are also indicated in the diagram.

Transcription factor (TF) activity in fibrotic microHOs and in NASH-induced cirrhotic human liver tissue was examined by analysis of scRNA-Seq data using the decoupleR method (45). Multiple TFs were activated in fibrotic microHOs and in cirrhotic liver tissue. Among the clusters in microHOs, MyoF_T1 and Cho3 cells exhibited the widest spectrum of TF activation (Figure 5A). Of interest, SMAD4, STAT1, and JUN activity was increased in MyoF_T1 and Cho3 cells after TGFB treatment (versus control, Figure 5, B and C). These TFs were also activated in cirrhotic NASH liver tissue, and liver myofibroblasts displayed the highest level of activation of these TFs (versus vascular smooth muscle or HSCs, Figure 5, D and E). PDGFB and TGFB are well-established drivers of liver fibrosis (46). TGFRB activation leads to activation of SMAD signaling pathways (47, 48), PDGFR cross-linking activates the STAT3 pathway (49, 50), and both of these pathways have well-known roles in promoting hepatic fibrosis (50). However, other pathways, which are jointly activated by these profibrotic agents, could provide new targets for antifibrotic drugs. Both TGFBR1 (47, 48, 51) and PDGFR (52, 53) activate the p38 MAPK and PI3K/AKT/mTOR intracellular signaling pathways, which are also pro-fibrotic (Figure 5F). Of relevance, several TFs in the p38 pathway were activated in MyoF_T1 in fibrotic microHOs (JUN, AP1, FOS) and in the myofibroblasts in cirrhotic human liver (JUNB, FOSB) (Figure 5, A and D). Hence, we hypothesized that effective antifibrotic agents could be produced by targeting pathways (i.e., p38 or PI3K/Akt/mTOR) that are jointly activated by TGFBRs and PDGFRs.

### Pharmacologic characterization of pro-fibrotic signaling pathways.

**Figure 6:**
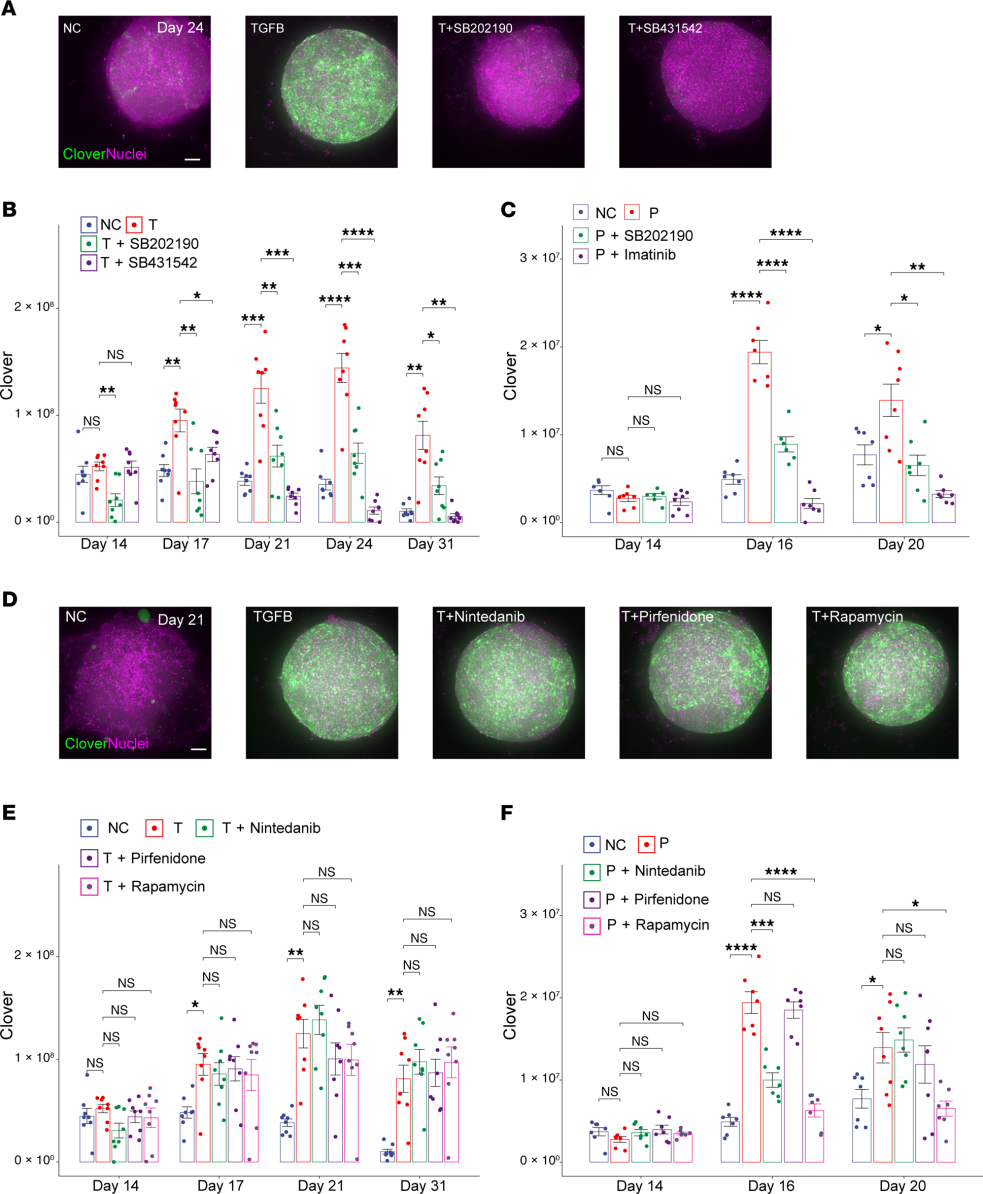
A p38 MAPK inhibitor targets both TGFB and PDGFB pathways. (**A**–**C**) TGFB- or PDGFB-induced fibrosis in microHOs is inhibited by a p38 MAPK inhibitor. (**D**–**F**) PDGFB-induced fibrosis is inhibited by pirfenidone or rapamycin. (**A** and **D**) MIP images of *Z*-stack sections obtained from microHOs treated with no addition (NC), or 50 ng/mL TGFB + 10 μM of the indicated drugs. Scale bar, 100 μm. Clover expression is green, and nuclei stained with Hoechst 33342 are purple. (**B**, **C**, **E**, and **F**) The number of COL1A1^+^ cells within a microHO was serially measured on days 14 through 31. Each dot represents a measurement made on a microHO, the thick line is the median of 8 microHOs that were assessed, and the box plot shows the 25% to 75% range for all measurements per condition. When only GFs were added, the *P* values were relative to the NC, and the significance indicators are *, *P* < 0.05; **, *P* < 0.01; ***, *P* < 0.001; or ****, *P* < 0.0001. When GFs and inhibitors were added, the *P* values were calculated relative to the PDGFB- or TGFB-treated microHOs (without added drug). A 2-way ANOVA indicates that drug treatment and time are 2 variables that have a significant interaction on the fluorescence measurements (Supplemental Table 9).

Although the antifibrotic effect of receptor tyrosine kinase inhibitors was growth factor dependent, other signaling pathways jointly activated by TGFBRs and PDGFRs could also be essential for fibrosis. Therefore, we examined whether coadministration of PI3K/AKT/mTOR (rapamycin) or MAPK pathway (SB202190) inhibitors with PDGFB or TGFB could reduce fibrosis. Coadministration of 10 μM SB202190 — a highly selective p38α and β kinase inhibitor (54) — inhibited both TGFB- and PDGFB-induced fibrosis in microHOs (Figure 6, A–C, and Supplemental Figure 3C). Thus, p38 MAPK pathway activity is essential for either PDGFB- or TGFB-driven fibrosis. Rapamycin partially inhibited PDGFB-induced fibrosis but did not inhibit TGFB-induced fibrosis (Figure 6, D–F). This result indicates that the downstream part of the PI3K/AKT/mTOR pathway was not essential for TGFB-induced fibrosis, but it could be required for PDGFB-induced fibrosis.

This microHO system could also be used to test the potential antifibrotic efficacy of candidate medications for liver fibrosis. For example, nintedanib and pirfenidone are the only 2 FDA-approved drugs for treatment of pulmonary fibrosis (55). Both inhibit multiple tyrosine kinases and have been shown to reduce lung fibrosis in animal models (1, 56) and in patients with interstitial lung fibrosis (57–61). Although the mechanism for their antifibrotic effect is unknown, it has been suggested that they could also be used to treat liver fibrosis (50). However, coadministration of 10 μM pirfenidone or 10 μM nintedanib did not inhibit the TGFB-induced fibrosis in microHOs. While pirfenidone did not inhibit PDGFB-induced fibrosis, nintedanib delayed the onset of PDGFB-induced fibrosis in microHOs (Figure 6, D–F). Of note, these concentrations were >10-fold above the nintedanib concentration that inhibited PDGFB-stimulated cellular proliferation (IC_50_ 64 nM) and PDGFR autophosphorylation (IC_50_ 22–39 nM) (62) and of the pirfenidone concentration (1 μM) that inhibited proliferation and *TGFB* mRNA expression (63) in cultured human fibroblasts. The partial inhibitory effect of nintedanib on PDGFB-induced fibrosis is consistent with its inhibition of the PDGFB receptor tyrosine kinase activity (IC_50_ 60 nM) (64).

**Figure 7:**
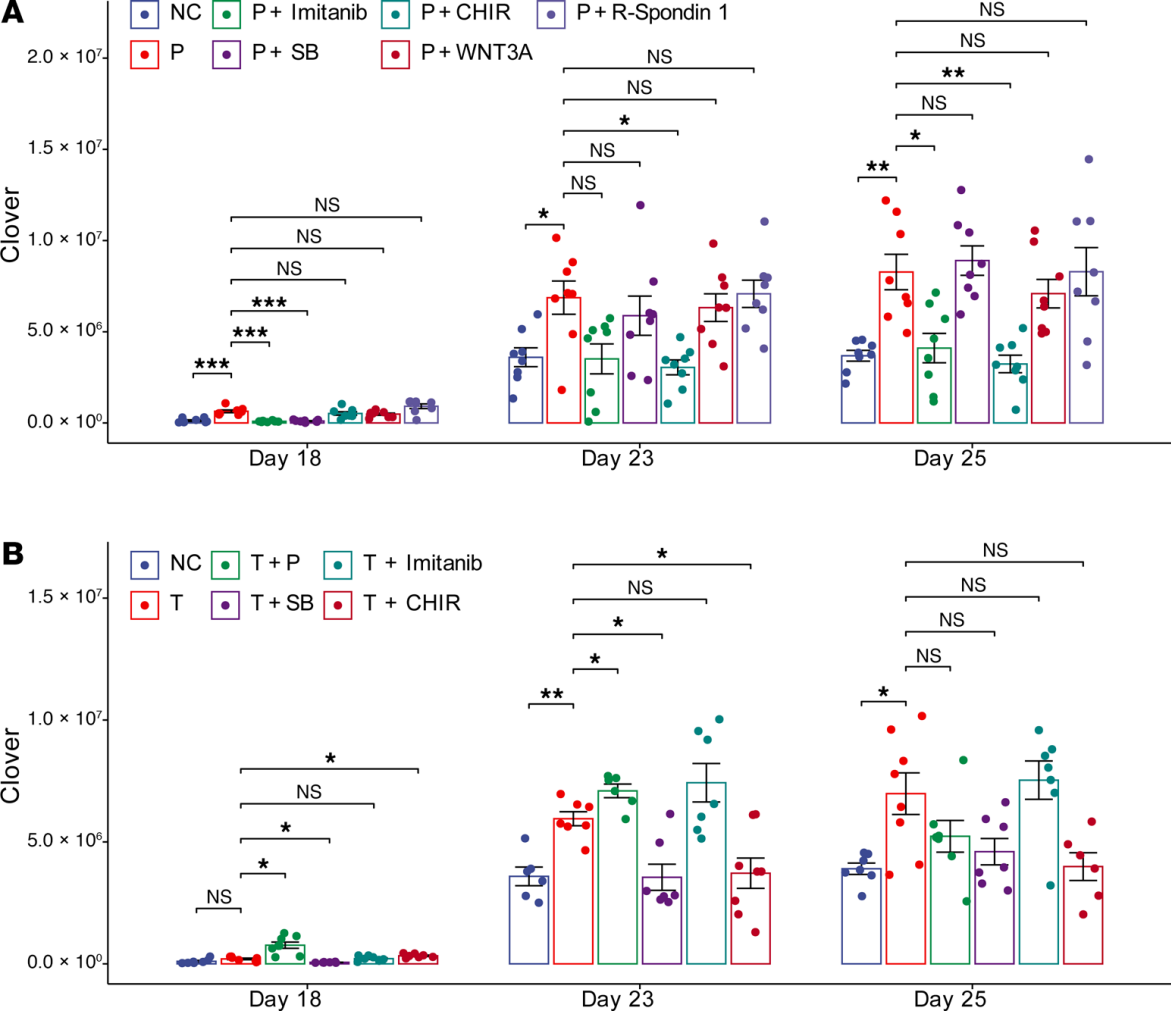
GSK3β inhibitors block TGFB- and PDGFB-induced fibrosis. (**A**) The PDGFB-induced increase in COL1A1^+^ cells is blocked by PDGFRβ and GSK3β inhibitors. *COL1A1*-P2A-Clover HOs were formed by adding 10,000 cells from day 9 organoid cultures to each microwell. Then, nothing (NC), 50 ng/mL PDGFB (P), or 50 ng/mL PDGFB with 10 μM imatinib, 10 μM SB431542 (SB), 10 μM CHIR99021 (CHIR), 100 ng/mL Wnt3a, or 100 ng/mL R-spondin 1 was added to each microHO on day 13. (**B**) The TGFB-induced increase in COL1A1^+^ cells is blocked by TGFBR1 and GSK3β inhibitors. Nothing (NC), 50 ng/mL TGFB (T), 50 ng/mL TGFB with 50 ng/mL PDGFB (T+P), or 50 ng/mL TGFB and 10 μM imatinib, 10 μM SB431542 (SB), or 10 μM CHIR99021 (CHIR) was added to each microHO on day 13. microHO fluorescence, which indicates the number of COL1A1^+^ cells, was serially measured on days 18 through 25. Each dot represents a measurement made on a microHO, the thick line is the median for 8 microHOs that were assessed, and the box plot shows the 25% to 75% range for the measurements. In the panels, *, *P* < 0.05; **, *P* < 0.01; or ***, *P* < 0.001 for the measurement relative to the value in the NC; when GFs or inhibitors were added, the *P* values were calculated relative to those of the PDGFB- or TGFB-treated cultures. A 2-way ANOVA indicates that drug treatment and time are 2 variables that have a significant interaction on the fluorescence measurements (Supplemental Table 9).

Wnt/β-catenin signaling contributes to the pathogenesis of liver fibrosis (65, 66), which led to the suggestion that it could be targeted by antifibrotic therapies (67). Since glycogen synthase kinase 3β–mediated (GSK3β-mediated) phosphorylation of β-catenin targets it for ubiquitin-dependent proteasomal degradation, GSK3β inhibitors stabilize β-catenin and promote the transcription of β-catenin target mRNAs (68, 69). Therefore, we examined the effect that coadministration of a Wnt/β-catenin pathway activator (Wnt3a) or of a GSK3β enzyme inhibitor (3 μM CHIR99021), which has been shown to activate the Wnt/β-catenin pathway (70), had on fibrosis in microHOs. Wnt3a coadministration had a minimal effect on the extent of PDGFB- or TGFB-driven fibrosis (Supplemental Figure 4). The GSK3β inhibitor by itself did not increase COL1A1^+^ cells in the organoids (Supplemental Figure 4). However, coadministration of the GSK3β inhibitor with either PDGFB or TGFB blocked fibrosis in microHOs (Figure 7 and Supplemental Figure 3B). Of importance, cell viability in microHOs was not reduced by addition of TGFB or by adding the GSK3β or TGFBR1 inhibitors (Supplemental Figure 15). Our results indicate that p38 MAPK or GSK3β kinase inhibitors can block liver fibrosis irrespective of whether it is driven by PDGFB or TGFB.

**Figure 8:**
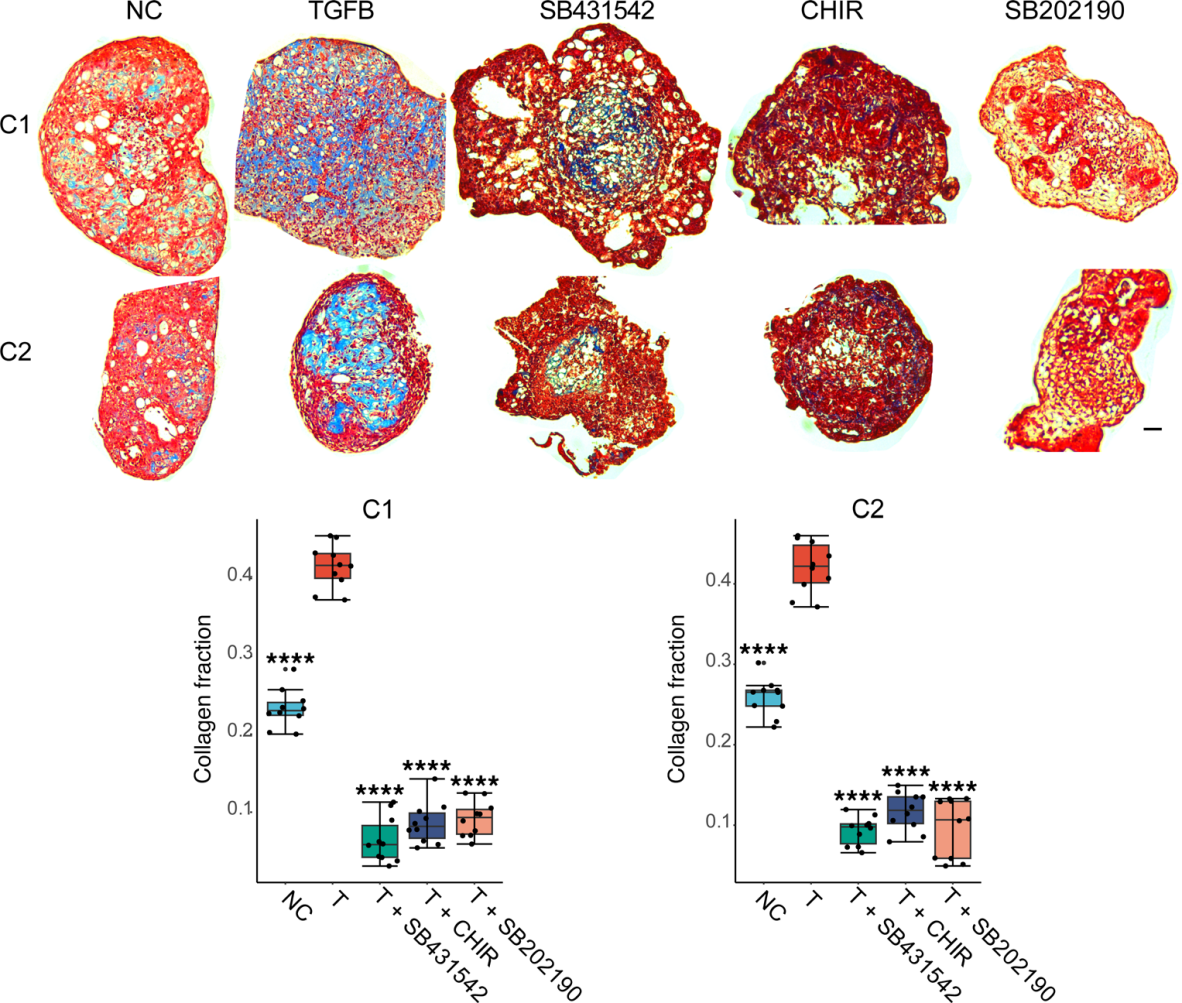
Antifibrotic drugs inhibit fibrosis in microHOs produced from iPSCs of different genetic backgrounds. microHOs produced from iPSC lines generated from 2 donors (C1, C2) (10) were treated with no addition (NC), TGFB (T) (50 ng/mL), or TGFB and 1 of the following drugs on day 13: TGFBR inhibitor (10 μM SB431542), GSK3β inhibitor (3 μM CHIR), or p38 inhibitor (10 μM SB202190). The microHOs were analyzed by trichrome staining on day 21. (**A**) Images of trichrome-stained, TGFB-treated microHOs show a marked increase in collagen-rich connective tissue (blue-stained regions) relative to control (NC) microHOs, which only had a thin layer of connective tissue. The TGFB-induced increased in collagen was markedly inhibited by addition of the TGFBR, GSK3β, or p38 inhibitors. The scale bar: 50 μm. (**B**) Box plots show the area in microHOs that received indicated treatments (*n* = 6–9 per group) occupied by collagen (collagen fraction). The means for each group were compared using a 1-way ANOVA and Tukey’s posttest: ****, *P* < 0.0001 (for T vs. NC or T vs. T+drug).

To determine if the antifibrotic effects of the TGFBR1, GSK3β, or p38 inhibitors was dependent upon the genetic background of the *COL1A1*-P2A-Clover line, we examined their antifibrotic effect in microHOs generated from iPSC lines (C1, C2) prepared from 2 other donors with different genetic backgrounds (10). As visualized by trichrome staining, TGFB induced a marked increase in collagen-rich connective tissue in the C1 and C2 microHOs (*P* < 0.001) that was markedly inhibited by addition of TGFBR1, GSK3β, or p38 inhibitors (*P* < 0.001) (Figure 8). This result indicates that the antifibrotic effect of these drugs was independent of the genetic background of the iPSCs used to produce the HO.

## Discussion

We demonstrate that microHOs and the live-cell imaging system developed here can be used to characterize the biochemical signals and intracellular signaling pathways that drive liver fibrosis and for assessing potential antifibrotic therapies. The TGFB- and PDGFB-induced fibrosis in microHOs was shown to resemble human fibrotic liver disease based upon the formation of thick collagen fibers (SHG results), and these agents increased collagen cross-linking (4-OHPro measurement) and collagen deposition (trichrome staining) in microHOs. Transcriptomic comparisons demonstrated that the transcriptomes of microHO myofibroblasts resembled the myofibroblasts in fibrotic human liver tissue. Of the agents tested — which included cytokines, growth factors, a hedgehog agonist, and Wnt ligands — only PDGFB and TGFB had a strong pro-fibrotic effect in microHOs. For several of the cytokines and growth factors tested, the absence of a pro-fibrotic effect in the organoid could be due to the absence of an immune system or of other cell types that are required for their pro-fibrotic effect. However, a key finding emerging from this study is that the antifibrotic efficacy of PDGFR and TGFBR1 tyrosine kinase inhibitors was dependent upon the factor driving the fibrosis, and this could explain why it has been so difficult to develop liver fibrosis therapies. When fibrosis is driven by PDGFRβ/STAT3 pathway activation — as occurs in ARPKD liver fibrosis (11) or in murine models of lung and bone marrow fibrosis (71) — PDGFR inhibitors exhibit antifibrotic efficacy but are less effective when the fibrosis is driven by other mechanisms. There are organ-specific and species-specific differences in the mechanisms mediating tissue fibrosis (9), and different types of liver injury produce different patterns of liver fibrosis in humans (72). Consistent with these differences, microHO data indicate that at least 1 (and possibly both) of the FDA-approved drugs for treatment of lung fibrosis may not be effective for liver fibrosis. It is also noteworthy that a combination treatment of PDGFR (imatinib) and TGFBR1 (galunisertib) (73) inhibitors was more effective than blockade of either pathway alone in a murine radiation-induced lung fibrosis model (74). Hence, to optimally treat liver fibrosis, therapies may have to be adjusted based upon the pathogenic driving factors, which may vary in different patients or in response to different inciting causes. There is already evidence that patients with diabetes can be subdivided into distinct subgroups based upon clinical features and biomarker results (75), and efforts are underway to optimize treatment selection for the different types of patients with diabetes (76). Just as in other diseases, application of the principles of precision medicine (i.e., using genetic or genomic information to optimize treatment selection) (77) could enable therapies for liver fibrosis to be successfully developed.

Another key finding was that GSK3β and p38 MAPK inhibitors potently blocked the fibrosis induced by TGFB or PDGFB. The GSK3β inhibitor effect was unexpected, since modulating Wnt/β-catenin signaling has had variable effects on liver fibrosis in mouse models (78, 79); in a murine bile duct ligation model, GSK3β inhibitor administration increased the extent of liver fibrosis (80). As discussed in Supplemental Data 2, there are multiple nodes where the TGFB, Wnt/β-catenin, and p38 MAPK pathways interact. These interactions could explain how a GSK3β inhibitor could block TGFB-induced fibrosis, but the mechanism for the GSK3β inhibitor effect on PDGFB-induced fibrosis is less clear. Nevertheless, our results indicate that the p38 MAPK pathway, which is activated by both TGFBRs and PDGFBRs, plays a key role in fibrosis. Although there were no prior data demonstrating that it had an effect on liver fibrosis, SB202190’s ability to inhibit fibrosis in microHOs is consistent with prior studies showing that this agent inhibited the development of renal interstitial (81) and corneal (82) fibrosis in animal models, and it blocked the conversion of human corneal fibroblasts into myofibroblasts in vitro (83). However, additional studies will have to be performed to characterize the mechanism(s) by which the Wnt/β-catenin and p38 MAPK pathways, along with the SMAD and STAT3 pathways that are also activated by TGFBRs and PDGFBRs, jointly contribute to liver fibrosis. Moreover, microHOs can also be used in conjunction with other recently developed genomic methods to provide a deeper understanding of the mechanisms mediating liver fibrosis (84). In summary, live-cell imaging of human microHOs has identified GSK3β and p38 MAPK inhibitors as potential new therapies for liver fibrosis, and it is likely that other new therapies and their mechanism(s) of action could subsequently be identified using this system.

## Methods

Further information can be found in Supplemental Methods.

### Chemicals.

The growth factors, drugs, antibodies, and other staining reagents are shown in Supplemental Tables 1 and 2.

### iPSCs and HO generation.

The human iPSC line (C3) used in this study was prepared as previously described (10). The biopsy sample used to generate this iPSC line was obtained according to a protocol (number 10368) approved by the Institutional Review Board at Stanford. The CRISPaint (13) plasmid (pCRISPR-HOT-Clover-BlastR) was obtained from Addgene (plasmid 138569; http://n2t.net/addgene:138569). A clover expression cassette (P2A-Clover) was inserted at the COOH terminus of the endogenous *COL1A1* gene of the iPSC line without a STOP code using a sgRNA (TTGGGATGGAGGGAGTTTAC). After blasticidin selection, colonies obtained from single cells were picked and genotyped for the correct in-frame insertion using forward (actcccacgtggtaatgccc); and reverse (cttcagggtcagcttgccg) primers. iPSCs were differentiated into HOs via culture in a series of media containing different growth factors using methods (10) that are described below.

### SHG microscopy.

SHG (collagen fibers) images from day 21 control, PDGF-treated, and TGFβ1-treated HOs were collected on a multimodal nonlinear optical microscope setup, consisting of a modified inverted confocal microscope (Nikon, Ti2-E with C2 scanner and 60× water immersion objective, NA = 1.27) and a picosecond-pulsed laser source (APE picoEmerald S, 2 ps pulse width, 80 MHz repetition rate, and 10 cm^–1^ bandwidth). As previously described (10), SHG data collection was made in the epi-direction with 2 narrow bandpass filters (Semrock FF01-400/12, Thorlabs MF390-18) and 1 shortpass filter (Thorlabs FESH0500). For the quantitative analyses, the area of the collagen fibers (identified by Otsu thresholding) was calculated for each SHG image and divided by the total sample area, forming the collagen area fraction (in %). To determine the diameter of the collagen fibers, we evaluated the local thickness of the fibers by applying the ImageJ Local Thickness routine (see the Analyze menu in Fiji) (85) to the SHG data. The volume fraction of thick collagen fibers (diameter ≥ 3 μm) was estimated by dividing the number of pixels represented by local thickness values ≥ 3 μm by the total number of pixels of the stack. To test whether the volume fractions obtained for control and TGFβ1- or PDGF-treated organoids were significantly different, the Welch’s *t* test was applied (*P < 0.05 and **P < 0.01).

### microHO generation.

To produce microHOs, 50%~60% confluent iPSC cultures were switched to an endoderm differentiation medium that consisted of DMEM/F12 + ITS (Gibco) supplemented with 0.1 mM nonessential amino acids, 1 mM pyruvate, and 2 mM l-alanyl-l-glutamine dipeptide (GlutaMAX, Gibco). On days 1 to 2, 100 ng/mL Activin-A (PeproTech), 10 ng/mL BMP4 (PeproTech), 100 ng/mL bFGF (PeproTech), 3 mM CHIR99021 (Selleckchem), and 10 μM LY294002 (Selleckchem) were added this medium. On day 3, 100 ng/mL Activin-A and 100 ng/mL bFGF were added the definitive endoderm differentiation medium. From days 4 to 9, 20 ng/mL FGF10 (PeproTech) and 20 ng/mL BMP4 were added to the hepatoblast medium, which consisted of Advanced RPMI 1640 Medium (Gibco) that was supplemented with GlutaMAX and ITS. After day 9, the hepatoblasts were dissociated to single cells in Accutase (Invitrogen) medium with 10 μM Y-27632 (Santa Cruz Biotechnology). Then, 1,000 to 5,000 cells/well were reaggregated in low-cell-adhesion Nunclon Sphera 96-well microplates (Thermo Fisher Scientific) that contained serum-free HO growth and differentiation medium, which consisted of William’s E medium supplemented with 0.1% polyvinyl alcohol (MilliporeSigma), 0.1 mM nonessential amino acids, 1 mM pyruvate, 2 mM GlutaMAX, 10 mM Y-27632, 100 ng/mL EGF, 10 ng/mL HGF, 10 μM dexamethasone, and 10 μM hydrocortisone (MilliporeSigma). On day 13, 50 ng/mL of TGFβ1 (PeproTech, 100-21; or Sinobiological, 10804-HNAC) or 50 ng/mL of PDGFB (Miltenyi Biotec, 130-108-163; or PeproTech, 100-14B) was added to each microwell.

### microHO analysis.

For COL1A1^+^ and Hoechst 33342 (Invitrogen, H3570) fluorescence measurements, 3D, stacked images were captured from day 14 to day 31 cultures using a Molecular Devices ImageXpress Micro Confocal system. For confocal imaging, 20 to 40 planes crossing the whole organoid at 20 μm intervals were captured using a 10× 0.45 Plan Apo 4 mm working distance objective with a 60 μm pinhole. For each 10× confocal *Z*-stack field (enough to cover 1 whole organoid), MIPs were generated from all the acquired *Z*-planes (the number planes ranged from 20 to 40 depending on the batch size). MIPs from each channel belonging to the same well were used for organoid segmentation and feature extraction by applying the machine learning–based Trainable Weka Segmentation (86) plug-in from Fiji (87). Each individual organoid edge was automatically segmented based on the nuclei signal (Hoechst 33342). Cell numbers were determined using the Find Maxima function in a defined area of an organoid after segmentation. *COL1A1*-P2A-Clover^+^ cells were segmented based on fluorescence. For training purposes, each analyzed element in every image (total 12 images) was used to train the classifiers by manually labeling all positive spots. Then, for experimental image analysis, the saved classifier was used to generate the probability map for each image. Segmented areas were then isolated, thresholded, and binarized, and the Integrated Density was calculated from segmented area. An unpaired 2-tailed *t* test was used to test whether the measurements were significantly different between each comparison group.

### Immunoblotting.

iPSC and day 9 hepatoblast cultures and day 21 control, PDGFB-treated, or TGFB-treated HOs (*n* = 16 per condition) were lysed in RIPA buffer. The lysates were analyzed by PAGE on a 4%–15% Tris-glycine gel. After blotting onto a membrane, the membranes were incubated with a 1:2,000 dilution of an anti-GFP antibody (Clontech 632381, clone JL8) for detection of Clover protein, and the secondary antibodies were IRDye 680 and 800 (LI-COR Biosciences, catalog 925-32210 and 925-68021).

### scRNA-Seq.

Control, PDGFB-induced, or TGFβ1-induced microHOs (*n* > 30 per group) were harvested from multiple independently prepared sets of cultures, and single-cell suspensions were prepared by protease digestion as described (10). The single-cell suspensions were visually inspected under a microscope. Cells were counted using a Scepter 2.0 Handheld Automated Cell Counter (EMD Millipore), then resuspended in PBS with 0.01% BSA. scRNA-Seq libraries were prepared by using the split-pool scRNA-Seq method (Evercode WT Mini v2, Parse Biosciences) according to the manufacturer’s instructions. In brief, approximately 5,000 cells from each group were split into 3 wells for the first round of sample barcoding, and the final pooled cells were divided into 2 equal libraries for sequencing. The expression matrix was generated using the Parse Biosciences analysis pipeline. A total of 38,716 features that were generated from 13,160 cells were generated from 4 experimental batches, and 57, 778 cells were recovered that passed quality control for processing of the scRNA-Seq data.

### Flow cytometry.

iPSCs were dissociated into single cells by incubation with 5 μM EDTA (Invitrogen) in PBS; hepatoblasts were dissociated using Accutase (Gibco). Single-cell suspensions were prepared from microHOs by protease digestion as described (10). Flow cytometry was performed using a BD Accuri C6 flow cytometer using the conjugated antibodies listed in Supplemental Table 2, and the data were analyzed using FCS Express (De Novo Software). A density plot of forward scatter height versus forward scatter area was used to exclude doublets.

### scRNA-Seq data analysis.

The scRNA-Seq data were imported into Seurat (88) for the subsequent analysis steps. (a) Cells with unique gene counts of <200 or where the percentage of mitochondrial mRNAs was >10% were removed. (b) A total of 2,000 variable genes were identified using the default settings. Four batches of data were integrated by standard processing methods using the Seurat integration function. Unwanted sources of variation were removed by regression analysis, which was performed to mitigate the effect of signals caused by mRNAs with unique molecular identifiers and mitochondrial expression. (c) A total of 10 principal components were used to construct the shared nearest neighbor graph, and the parameter regulating the resolution of the FindClusters program was set to 0.5. This resulted in the identification of 11 unique clusters for all 57,778 cells. To identify the cell type of each cluster, differentially expressed genes for the predefined cell types were computed using the FindAllMarkers function within the Seurat Package with the following parameters: only.pos = TRUE, min.pct = 0.25, logfc.threshold = 0.25. Seurat then identified the differentially expressed genes using the nonparametric Wilcoxon rank sum test. The top 100 differentially expressed genes were used for the GO biological process analysis, which was performed using the clusterProfiler for GO overrepresentation analysis (89).

### Module score calculation.

Module scores were calculated to assess the relationship between the transcriptomes of microHO clusters and the different types of cells in human liver tissue. scRNA-Seq data obtained from normal and cirrhotic human liver tissue (GSE136103) (90) was used to identify the different types of liver cells. Differentially expressed genes were calculated for each of the clusters in microHO (MyoF_T1, MyoF_P, Mes2) and human liver tissue (Myofibroblast, HSC, VSMCs, and Meso), using the FindMarkers function of the R package in Seurat (91). Then, the gene signatures used for determining the module score were selected based on the intersection between the marker genes in microHOs and those in the cell types in human liver. There were 53, 84, and 31 shared genes that were used to calculate the module scores for MyoF_T1 versus MyoF in liver, MyoF_P versus liver MyoF, and Mes2 versus liver HSC, respectively. For comparisons of the scores obtained with VSMCs, HSCs, myofibroblasts, and mesothelial cells, a 1-way ANOVA was used to compare the means of the measurements for the 4 groups. The Tukey multiple comparison of the means test was then used to determine whether there was a significant difference between the means of all possible comparisons.

### ANOVA calculations.

The 2-way ANOVA was used to compare the means of the COL1A1:Clover signal (calculated as area × mean intensity [IntDen]) between the different drug treatment groups and time points, while assessing interaction effects between the treatment groups and time. The 2-way ANOVA was performed using the aov function of the R stats package, and both groups and time points were analyzed as categorical independent variables. The null hypothesis was that there is no difference in the means of the different treatment groups or time points and that the treatment groups and time points do not interact in any way. Since the 2-way ANOVA of the data shown in Figure 2, F and G; Figure 6, B and C; and Figure 7, A and B, use separate datasets, a correction for multiple testing is not needed.

### RNA velocity, CytoTRACE2, cell-cell communication, and TF activity analysis.

RNA velocity analysis infers cell state by measuring the ratio of unspliced to spliced mRNA transcripts, which provides information about the transcriptional activity of a gene and its direction. The transcript assignment file (tscp_assignment.csv.gz from the Parse pipeline output directory) contains the splicing information for each transcript identified in a Parse assay. The splicing information is used to generate the splice matrices that are required to run scVelo (41). The Anndata file, which is required for scVelo, was generated using the Seurat object that contains the metadata and splicing matrix. RNA velocity was estimated with the stochastic model (using second-order moments). Cellular potency categories and the absolute developmental potential were assessed with CytoTRACE 2 (42) using the scRNA-Seq data. The predicted potency scores provide a continuous measure of developmental potential; they range from 0 (differentiated) to 1 (totipotent). The raw count from Seurat object is used to directly compute CytoTRACE score.

The R toolkit CellChat v2 was used for inference, visualization, and analysis of cell-cell communication based upon the scRNA-Seq data (92). The All CellChatDB, except for the “Non-protein signaling” component, was used for analysis of cell-cell communication. The R implementation of decoupleR (45) was used to extract biological activities from the CollecTRI database. CollecTRI is a comprehensive resource containing a curated collection of TFs and their transcriptional targets compiled from 12 sources (93). The interactions are weighted based upon their mode of regulation (activation or inhibition).

### Analysis of drug effect in C1 and C2 microHOs.

The C1 and C2 iPSC lines were generated and characterized as previously described (10) and used to prepare microHOs as described above. Trichrome staining of day 21 microHOs was performed according to the manufacturer’s instructions using Masson’s 2000 trichrome stain (American Mastertech). Image segmentation and quantitative measurement of collagen-rich areas were performed using our previously described methods (11). In brief, Fiji (2.1.0) implementation of ImageJ was used to quantify the areas of positive staining. The Trainable Weka Segmentation plug-in was used to train the classifiers and calculate the test experimental image. Statistical analysis was performed using a 1-way ANOVA and Tukey’s posttest.

### Statistics.

For analysis of whether the volume fractions obtained for control and TGFβ1- or PDGF-treated organoids in SHG images were significantly different, Welch’s *t* test was applied with cutoffs of *P* < 0.05 and *P* < 0.01. For analysis of the images of drug effect on microHOs, an unpaired 2-tailed *t* test was used. For comparing the effect of drugs on fibrosis in microHOs, a 2-way ANOVA was used to compare the means of the COL1A1:Clover signal (calculated as area × mean intensity [IntDen]) between the different drug treatment groups and time points, while assessing interaction effects between the treatment groups and time. Statistical analysis of drug effects on fibrosis in C1 and C2 HOs was performed using a 1-way ANOVA to assess the overall differences between group means, and Tukey’s posttest was then used for pairwise comparisons to identify groups with significant differences in their means.

### Study approval.

The iPSCs were prepared according to a protocol (no. 10368) that was approved by the Institutional Review Board at the Stanford University School of Medicine.

### Data availability.

All raw and processed scRNA-Seq data were deposited in the National Center for Biotechnology Information Gene Expression Omnibus and are available under accession GSE228214. The macro script used for microHO analysis is available upon request. A supplemental file with Supporting Data Values is available.

## Author contributions

YG, SR, AH, and PKJ generated experimental data; ZF, YG, PKJ, AE, MW, WR, SCH, and GP analyzed data; and the paper was written by YG and GP with input from all authors.

## Supplementary Material

Supplemental data

Supporting data values

## Version 1 12/10/2024

In-Press Preview

## Version 2 01/23/2025

Electronic publication
