# Supplementary material for: Live-cell imaging of human liver fibrosis using hepatic micro-organoids
Source: JCI Insight. 2024 Dec 10;10(2):e187099. doi: 10.1172/jci.insight.187099 (PMC11790020; doi:10.1172/jci.insight.187099)
Supplement: Supplemental data [file jciinsight-10-187099-s079.pdf]

Supplemental Information

Live Cell Imaging of Human Liver Fibrosis using Hepatic Micro-Organoids

Supplemental note 1: *Supplemental Note 1*: Gene Set Enrichment Analysis (**GSEA**) (1) was used to investigate whether microHOs fibrosis resembled two commonly occurring forms of human liver fibrosis. To do this, genes whose expression levels were increased in MyoF_T1 (n=527) or MyoF_P (n=1716) relative to Mes2 were used to form myofibroblast-specific gene expression signatures. GSEA calculates a normalized expression score (**NES**), which indicates whether the signature genes were enriched in fibrotic or non-fibrotic liver tissue. GSEA was first performed using expression data obtained from early (non-fibrotic) stage 1 and late (fibrotic) stage 4 NASH liver tissue (GSE13525 (2)). Liver fibrosis develops as NASH liver disease advances; myofibroblast activation is key to its pathogenesis (3-5); and the extent of liver fibrosis is the major determinant of NASH outcome (6, 7). GSEA results indicate that the MyoF_T1 (NSE 1.56; false discovery rate (FDR) 0.0178) gene expression signature was very strongly associated with stage 4 NASH, but not with early (NES -0.85 FDR 1) stage 1 NASH liver (**Fig. S12A, Table S9)**). The MyoF_T1 (NES 1.71, FDR 0.005) signature was also associated with liver fibrosis caused by hepatocellular carcinoma, whereas it (NES -1.32, FDR 0.17) was not associated with non-fibrotic hepatocarcinoma liver tissue (GSE6764 (8)) (**Fig. S12B**). In contrast, The MyoF_P signature had a positive (but not statistically significant association) with stage 4 NASH (NES 0.45, FDR 0.14) and HCC-induced liver fibrosis (NES 0.36 FDR 0.06). Thus, GSEA analyses indicate that the MyoF_T1 signature in microHOs resemble those in two commonly occurring types of human liver fibrosis.

Supplemental note 2: *Interactions between the TGFβ, Wnt/β-catenin and p38 MAPK pathways*. The TGFβ1 and Wnt/β-catenin pathways have several interaction points. (i) TGF-β significantly enhances hepatic WNT-5A expression *in vivo* and in myofibroblasts *in vitro* (9). (ii) The interdomain regions of SMAD transcription factors have two residues that are phosphorylated by GSK3β in the nucleus, and their phosphorylation state regulates SMAD transcriptional activity and turnover (10). Hence, the ability of the GSK3β inhibitor to block TGFβ1-induced fibrosis in microHOs could (at least in part) be mediated by altering the phosphorylation state of these two sites, which could alter SMAD transcription factor activity. (iii) During development, receptor tyrosine kinase and Wnt/β-catenin pathway signals were shown to be integrated by the level of SMAD phosphorylation (11).

The Wnt/β-catenin and p38 MAPK pathways also interact at several nodes. (i) Wnt ligands have been shown to activate p38 MAPK activity (12). (ii) GSK3β phosphorylation by p38 MAPK regulates its kinase activity. MAPK kinases have a Thr–Gly–Tyr dual phosphorylation motif within their kinase activation loop, and both sites must be phosphorylated to fully activate the kinase. The MAP2K kinases MKK3 and MKK6 are the major upstream kinases for p38 activation (13). However, phosphorylation on Ser^{389} on GSK3β is a major mechanism for downregulating its kinase activity and p38α was shown to inactivate GSK3β by this mechanism: p38-mediated phosphorylation of Ser^{389} on GSK3β was shown to cause β-catenin accumulation and activation of the Wnt signaling pathway (12, 14). By this mechanism, p38 MAPK inhibitors could reduce Wnt pathway activation, which could prevent the development of a fibrosis that was dependent upon Wnt signaling.

Supplemental Methods

Immunohistochemistry. Fresh organoids were harvested and fixed in 4% paraformaldehyde for 30 minutes. All organoids were either stained directly or embedded in low melting point agarose (IBI Scientific, Dubuque, Iowa). The primary antibodies used for staining are listed in **Table S2**. The organoids were stained with the primary antibody, and permeabilized by incubation with 1% Triton X-100 (Sigma-Aldrich, St. Louis, MO) and 10% chicken serum (Jackson ImmunoResearch, Bar Harbor, MA) overnight. Then, secondary antibody-staining was performed using an Alexa Fluor labeled chicken anti IgG (H+L), which was cross-adsorbed with a secondary antibody in 10% chicken serum (Invitrogen, Pleasanton, CA).

4-hydroxyproline quantitation. iPSC and day 9 hepatoblast cultures and day 21 control, PDGF- or TGFβ-treated HOs (n=16 per condition) were isolated and snap frozen in liquid nitrogen. 4-hydroxy proline levels were measured using the Hydroxyproline Assay kit (Sigma, MAK008) according to the manufacturer’s instructions. In brief, HOs were homogenized in 100 μL of water and transferred to a pressure-tight polypropylene vial. Then, 100 μL of concentrated hydrochloric acid (HCl,12 M) was added, a cap was placed on tightly on the vial, and the sample was hydrolyzed at 120°C for 3 hours. The hydrolysate was centrifuged at 10,000 × g for 3

minutes. The clear supernatant was transferred and used for colorimetric assay of 4-hydroxyproline, which was performed according to the manufacturer's instructions.

Trichrome staining. Day 21 control, PDGF- or TGFβ- (+ drug) treated organoids were harvested, allowed to settle by gravity, and were embedded in low melting point agarose (IBI Scientific, Dubuque, Iowa). The embedded organoids were then processed by sectioning of the paraffin-embedded tissue to produce 10-micron tissue sections. To assess the amount of collagen in the organoids, trichrome staining of the tissue sections was performed using the MASSON'S 2000 TRICHROME STAIN KIT according to the manufacturer's instructions.

Gene signature expression analyses (GSEA). The myofibroblast-specific gene expression signatures for genes whose expression was upregulated in MyoF_T1 (n=527) or MyoF_P (n=1716) relative to Mes2 (Table S*) were identified using the '*FindMarkers*' function in '*Seurat*' (15) with the parameter 'min.pct' set at 0.25 (which indicates the minimum fraction of cells within a cluster that expressed a given gene); and the default Wilcoxon rank sum test was used to perform this analysis. Two publicly available gene expression datasets for NASH (GSE135251 (2)) and cirrhotic (GSE6764 (8)) liver were obtained from the Gene Expression Omnibus using the '*GEOquery*' as described (16). Cirrhotic liver tissue was obtained from 10 subjects undergoing liver resection for hepatocellular carcinoma at one of 3 US or European hospitals; and their liver tissues were classified as cirrhotic by the examining pathologist. The hepatocellular carcinoma samples (HCC) samples examined in this study were obtained from eight subjects whose liver was resected for HCC, but these specimens did not have fibrosis or cirrhosis according to the examining pathologist. The 10 normal liver tissues (used for comparison) were obtained from 10 subjects undergoing liver resection for other reasons at the same hospitals, and their liver tissue was classified as normal by the examining pathologist (8). For the NASH analysis, 216 liver biopsies were examined by histology, which generated the

fibrosis stage scores: 4 biopsies were classified as Stage 4, 48 biopsies were classified as Stage 1, and 46 as stage 0.

The GSEA for the myofibroblast signatures were calculated for the gene expression datasets generated from these samples using previously described methods (1); and 1000 permutations were used for significance assessment for each analysis. The Enrichment score (ES) reflects the degree to which the Myofibroblast gene set is overrepresented at the top or bottom of a ranked list of genes; the Normalized enrichment score (NES) was used to compare analysis results across gene sets; and the false discovery rate (FDR) was used to estimate the probability that a gene set with a given NES represents a false positive finding.

References

1. Subramanian A, Tamayo P, Mootha VK, Mukherjee S, Ebert BL, Gillette MA, et al. Gene set enrichment analysis: a knowledge-based approach for interpreting genome-wide expression profiles. *Proc Natl Acad Sci U S A.* 2005;102(43):15545-50.
2. Pfister D, Nunez NG, Pinyol R, Govaere O, Pinter M, Szydlowska M, et al. NASH limits anti-tumour surveillance in immunotherapy-treated HCC. *Nature.* 2021;592(7854):450-6.
3. Tsuchida T, and Friedman SL. Mechanisms of hepatic stellate cell activation. *Nature reviews Gastroenterology & hepatology.* 2017;14(7):397-411.
4. Sircana A, Paschetta E, Saba F, Molinaro F, and Musso G. Recent Insight into the Role of Fibrosis in Nonalcoholic Steatohepatitis-Related Hepatocellular Carcinoma. *International journal of molecular sciences.* 2019;20(7).
5. Marcher AB, Bendixen SM, Terkelsen MK, Hohmann SS, Hansen MH, Larsen BD, et al. Transcriptional regulation of Hepatic Stellate Cell activation in NASH. *Sci Rep.* 2019;9(1):2324.
6. Tanaka N, Kimura T, Fujimori N, Nagaya T, Komatsu M, and Tanaka E. Current status, problems, and perspectives of non-alcoholic fatty liver disease research. *World J Gastroenterol.* 2019;25(2):163-77.
7. Angulo P, Kleiner DE, Dam-Larsen S, Adams LA, Bjornsson ES, Charatcharoenwitthaya P, et al. Liver Fibrosis, but No Other Histologic Features, Is Associated With Long-term Outcomes of Patients With Nonalcoholic Fatty Liver Disease. *Gastroenterology.* 2015;149(2):389-97 e10.
8. Wurmbach E, Chen YB, Khitrov G, Zhang W, Roayaie S, Schwartz M, et al. Genome-wide molecular profiles of HCV-induced dysplasia and hepatocellular carcinoma. *Hepatology.* 2007;45(4):938-47.
9. Beljaars L, Daliri S, Dijkhuizen C, Poelstra K, and Gosens R. WNT-5A regulates TGF-beta-related activities in liver fibrosis. *Am J Physiol Gastrointest Liver Physiol.* 2017;312(3):G219-G27.

10. Macias MJ, Martin-Malpartida P, and Massague J. Structural determinants of Smad function in TGF-beta signaling. *Trends Biochem Sci.* 2015;40(6):296-308.
11. Fuentealba LC, Eivers E, Ikeda A, Hurtado C, Kuroda H, Pera EM, et al. Integrating patterning signals: Wnt/GSK3 regulates the duration of the BMP/Smad1 signal. *Cell.* 2007;131(5):980-93.
12. Bikkavilli RK, Feigin ME, and Malbon CC. p38 mitogen-activated protein kinase regulates canonical Wnt-beta-catenin signaling by inactivation of GSK3beta. *J Cell Sci.* 2008;121(Pt 21):3598-607.
13. Zarubin T, and Han J. Activation and signaling of the p38 MAP kinase pathway. *Cell Res.* 2005;15(1):11-8.
14. Thornton TM, Pedraza-Alva G, Deng B, Wood CD, Aronshtam A, Clements JL, et al. Phosphorylation by p38 MAPK as an alternative pathway for GSK3beta inactivation. *Science.* 2008;320(5876):667-70.
15. Butler A, Hoffman P, Smibert P, Papalexi E, and Satija R. Integrating single-cell transcriptomic data across different conditions, technologies, and species. *Nat Biotechnol.* 2018;36(5):411-20.
16. Davis S, and Meltzer PS. GEOquery: a bridge between the Gene Expression Omnibus (GEO) and BioConductor. *Bioinformatics.* 2007;23(14):1846-7.
17. Guan Y, Enejder A, Wang M, Fang Z, Cui L, Chen SY, et al. A human multi-lineage hepatic organoid model for liver fibrosis. *Nature communications.* 2021;12(1):6138.
18. Ramachandran P, Dobie R, Wilson-Kanamori JR, Dora EF, Henderson BEP, Luu NT, et al. Resolving the fibrotic niche of human liver cirrhosis at single-cell level. *Nature.* 2019;575(7783):512-8.

Table S1. Drugs and chemicals used in these studies.

CDCA	sigmaaldrich	C9377
Activin A Protein	sinobiological	10429-HNAH
Animal-Free Recombinant Human EGF	peprotech	AF-100-15
EGF	sinobiological	10605-HNAE
FGF4	peprotech	100-31
HGF	sinobiological	10463-HNAS
FGF10	sinobiological	10573-HNAE
FGF2 Protein, Human, Recombinant	sino	10014-HNAE
FGF10	Peprotech	100-26
Human PDGF-BB IS	miltenyibiotec	130-108-163
Human VEGF (165) IS	miltenyibiotec	130-109-383
Recombinant Human PDGF-BB (carrier-free)	biolegend	577302
Recombinant Human PDGF-BB	peprotech	100-14B
Recombinant Human PDGF-BB Protein, CF	rndsystems	220-BB-010In Stock
TGF beta 1 Protein, Human, Rhesus, Cynomolgus, Canine, Recombinant	sinobiological	10804-HNAC
Recombinant Human BMP-4	peprotech	120-05
Recombinant Human BMP-4 (carrier-free)	biolegend	795606
Recombinant Human/Mouse/Rat Activin A	r&d	338-AC-010
Recombinant Human FGF-Basic (146 a.a.)	Peprotech	AF-100-18C
Wnt3a	Peprotech	315-20
OSM	Peprotech	300-10
IL3 Protein, Human, Recombinant (His Tag)	sinobiological	11858-H08H
IL6	r&d	7270-IL-010/CF
IL-13 Protein, Human, Recombinant	sinobiological	10369-HNAC
IL-33 Protein, Human, Recombinant	sinobiological	10368-HNAE
IGF1	r&d	291-G1-200
R-Spondin 1 Protein	r&d	4645-RS
JAG-1 protein	StemRD	Cat#: JAG-1-pep-100
SB202190 (FHPI)	selleckchem	S1077
SB431542	Selleck	S1067
Sirolimus (Rapamycin)	medkoo	100766
Imatinib mesylate	medkoo	100470
DAPT (GSI-IX)	selleckchem	S2215
LDN-193189	selleckchem	S2618
SAG	selleckchem	S7779
IWR1-en	selleckchem	S7086
A-83-01	Santa Cruz Biotechnology	sc-203791
PI-103	Tocris	2930
PD0325901	Santa Cruz Biotechnology	sc-205427
CHIR99021	Tocris	4423
Wnt-C59	Tocris	5148
BIO	selleckchem	S7198
Thiazovivin	medchemexpress	HY-13257
Nintedanib	Tocris	7049
Pirfenidone	Tocris	1093
polyvinyl alcohol	sigma	P8136
Matrigel	BD	354234

Table S2. Antibodies and staining reagents used in these studies.

Name	Company	Catalog#	Dilution
CK8	abcam	ab53280	1/100 - 1/250
CK8	DSHB	TROMA-I	200
COL1	ABCAM	ab34710	1:100
GFP Tag Polyclonal Antibody	thermofisher	A-11122	200-2000
GFP Antibody (B-2)	scbt	sc-9996	50
Human Albumin Antibody	Bethyl	A80-129A	1:200 – 1:2,000
PDGFRB	eBioscienc	14-1402-82	100
PDGFRB	ABCAM	ab32570	100
PDGFRB	R&D	AF385-SP	100
PDGFRB	ll Signaling Technolo	3169T	100
LipidSpot™ Lipid Droplet Stains	biotium	#70069-T	
MitoSox Red	Invitrogen	M36008	
Alexa Fluor® 647 anti-human CD326 (EpCAM) Antibody	biolegend	324212	1:20

Cluster	Cell Type	NC (%)	PDGF (%)	TGFβ (%)
0	Mes1	20.7	20.5	20.2
1	MyoF_T1	1.9	1.5	37.5
2	Mes2	24.9	4.8	3.3
3	Cho1	17.6	14.7	1.3
4	Hep	12.8	12.3	5.2
5	MyoF_T2	3.6	7.07	16.2
6	Cho2	13.5	7.1	0.7
7	MyoF_P	0.8	20.1	1.0
8	Mes3	2.2	6.5	5.7
9	Mes4	1.9	4.4	3.0
10	Cho3	0.1	1.0	6.0

Table S3. The cell types and the percentage of the total number of cells in the 11 cell clusters identified by analysis of scRNA-Seq data in day 21 control, PDGF-, and TGFβ1-treated microHOs are shown. The transcriptomes of the 10 other cell clusters in the day 21 microHOs were defined using canonical markers and by comparison with the cells in control and cirrhotic human livers. Based upon the expression of canonical mRNAs and level of concordance between the cell cluster transcriptomes and the cell types identified in human liver, the microHOs have cholangiocytes (Cho1-3), hepatocyte (Hep), mesenchymal cell (Mes1-4) and myofibroblast (MyoF_T1-2, MyoF_P) clusters. A myofibroblast cluster (MyoF_T1) was far more abundant in the TGFβ1-treated microHOs than in control or PDGF-treated microHOs (37.5% of total vs <2%), while another myofibroblast cluster (MyoF_P) was more abundant in the PDGF-treated microHOs than in control or TGFβ1-treated microHOs (20% vs <2% of the total number of cells).

Table S4 is provided at the end of this file.

Table S5. The results of 1-way ANOVA analyses of the cell percentages calculated by analysis of the scRNA-Seq data for the five biological replicates from the control, PDGF- and TGFβ-treated microHO preps shown in Figure S8. The degrees of freedom (DF), the sum of the squares (Sum Sq) and mean squared (mean Sq) of the variance, calculated F-value, and the probability Pr(>F) from the ANOVA are shown. A Pr(>F) < 0.05 indicates that there was a significant effect of the indicated variable. There were significant differences in the % of MyoF_T1, Mes2, MyoF_P and Cho3 cells as indicated by the p-value: ***; 0.001, **; 0.01, *; 0.05. However, Cho3 abundance was $\leq$1% in all three types of microHOs.

	Df	Sum Sq	Mean Sq	F value	Pr(>F)	
Mes1	2	5	2.6	0.009	0.991	
Residuals	12	3599	299.9			
	Df	Sum Sq	Mean Sq	F value	Pr(>F)	
**MyoF_T1**	2	1259	629.7	6.046	**0.0153**	*
Residuals	12	1250	104.1			
	Df	Sum Sq	Mean Sq	F value	Pr(>F)	
**Mes2**	2	747.4	373.7	8.941	**0.00419**	**
Residuals	12	501.6	41.8			
	Df	Sum Sq	Mean Sq	F value	Pr(>F)	
Cho1	2	339.8	169.92	1.946	0.185	
Residuals	12	1047.8	87.32			
	Df	Sum Sq	Mean Sq	F value	Pr(>F)	
Hep	2	134.6	67.28	0.974	0.405	
Residuals	12	828.7	69.05			
	Df	Sum Sq	Mean Sq	F value	Pr(>F)	
MyoF_T2	2	145.5	72.73	2.739	0.105	
Residuals	12	318.7	26.56			
	Df	Sum Sq	Mean Sq	F value	Pr(>F)	
Cho2	2	169.3	84.63	2.754	0.104	
Residuals	12	368.7	30.73			
	Df	Sum Sq	Mean Sq	F value	Pr(>F)	
**MyoF_P**	2	425.9	213.0	3.988	**0.047**	*
Residuals	12	640.8	53.4			
	Df	Sum Sq	Mean Sq	F value	Pr(>F)	
Mes3	2	61.31	30.66	2.503	0.123	
Residuals	12	146.99	12.25			
	Df	Sum Sq	Mean Sq	F value	Pr(>F)	
Mes4	2	22.8	11.40	1.045	0.382	
Residuals	12	130.9	10.91			
	Df	Sum Sq	Mean Sq	F value	Pr(>F)	
**Cho3**	2	90.6	45.30	4.242	**0.0404**	*
Residuals	12	128.1	10.68			

Table S6. The gene symbols for 3 sets of genes whose mRNAs were used for calculation of the module scores shown in Figure 4G. (i) The MyoF_T1 mRNAs were derived from the intersection of the differentially expressed marker genes for MyoF_T1 versus Mes2 clusters. (ii) The MyoF_P mRNAs were derived from the intersection of differentially expressed marker genes for MyoF_P vs Mes2 clusters. (iii) The Mes2 mRNAs were derived from the intersection of the differentially expressed marker genes for Mes2 vs MyoF_T1 clusters.

	MyoF_T1			MyoF_P			Mes2
1	OGN		1	PTGDS		1	TM4SF1
2	LTBP2		2	LIFR		2	NPR3.00
3	LTBP1		3	OGN		3	TXNIP
4	IGFBP7		4	GPNMB		4	EPAS1
5	MMP2		5	LGALS3BP		5	ARHGAP29
6	DPYSL3		6	PLTP		6	INPP4B
7	ITGA1		7	THY1		7	MEF2C
8	COL4A2		8	MATN2		8	FRZB
9	COL4A1		9	ITGA1		9	ALDH1A1
10	GSN		10	LTBP1		10	RERG
11	IGFBP4		11	COL4A1		11	SYTL2
12	SERPINE2		12	COL4A2		12	PLA2G5
13	COL6A3		13	TIMP1		13	ANGPT1
14	TIMP1		14	SPARC		14	ARHGAP15
15	VCAN		15	VCAN		15	EBF1
16	COL1A2		16	FN1		16	NRP1
17	COL1A1		17	HGF		17	AKAP12
18	FN1		18	FBLN5		18	SLC40A1
19	BGN		19	GPC3		19	RAPGEF5
20	THY1		20	IGFBP3		20	EZR
21	FMOD		21	MGP		21	SLC2A3
22	PRSS23		22	PLXDC2		22	APOA2
23	SERPINA1		23	COL6A3		23	NFASC
24	CYP1B1		24	EDNRB		24	PDE1A
25	IGFBP6		25	DPT		25	ALB
26	ELN		26	PDGFRA		26	HMGB2

**27**	PDGFRA		27	IGFBP7		27	APOA1
**28**	SPARC		28	LMCD1		28	ZBTB16
**29**	F3		29	C7		29	HDAC2
**30**	PDLIM3		30	KRT18		30	DNAJC15
**31**	FBLN5		31	SERPINA1		31	NHSL2
**32**	ECM1		32	NR2F1			
**33**	DPT		33	MASP1			
**34**	FAP		34	FMOD			
**35**	S100A6		35	NR2F1-AS1			
**36**	NR2F1		36	MFAP4			
**37**	MFAP4		37	PLPP3			
**38**	ITGBL1		38	MIR99AHG			
**39**	SPON2		39	C3			
**40**	C3		40	RAPGEF5			
**41**	LMCD1		41	FGF7			
**42**	THBS1		42	PTN			
**43**	KRT18		43	IL6ST			
**44**	PRELP		44	MMP2			
**45**	A2M		45	PCDH9			
**46**	HSPB6		46	FAP			
**47**	CFH		47	THBS2			
**48**	SOD2		48	GPC6			
**49**	THBS2		49	S100A6			
**50**	PLTP		50	NRP1			
**51**	IFI6		51	COL1A2			
**52**	NR2F1-AS1		52	IGFBP6			
**53**	EMP1		53	PTGIS			
			54	SPON2			
			55	DPYSL3			
			56	HSPB6			
			57	ABCA8			

			58	SERPINE2			
			59	ARHGAP15			
			60	EMP1			
			61	F3			
			62	APOA2			
			63	PDLIM3			
			64	DNAJC15			
			65	APOA1			
			66	VCAM1			
			67	CFH			
			68	CYP1B1			
			69	THBS1			
			70	ITGBL1			
			71	ANK3			
			72	PRSS23			
			73	LTBP2			
			74	LAMA2			
			75	ELN			
			76	CFI			
			77	SOD2			
			78	CD44			
			79	BGN			
			80	DCN			
			81	COLEC10			
			82	PRELP			
			83	IGFBP4			
			84	A2M			

Table S7. One-way ANOVA and Tukey test for multiple comparisons of the module scores shown in Figure 4G. (**A**) The gene signatures for each cluster (Mes2, MyoF_T1, MyoF_P) were compared with four groups of human liver cells: vascular smooth muscle cells (VSMC); HSC; myofibroblasts; and mesothelia (Meso). The mean module score (Mean) and ratio of module score for each cell type relative to that with the highest mean (Fold) are shown. (**B, C**) Pairwise comparisons between HSC and three other types of human liver cells (VSMC, MyoF and Meso) produce significantly different results ($P<1\times10^{-10}$); and pairwise comparisons between MyoF and the three other types of human liver cells (VSMC, HSC and Meso) also produce significantly different results ($P<1\times10^{-10}$). Taken together, the ANOVA and pairwise comparison results indicate that the gene signature of myofibroblasts in liver is most similar to MyoF_T1 and MyoF_P in microHOs, and that the gene signature of HSC is most similar to Mes2.

Table S7A. The Mean of four liver cell types and different clusters and their relative Fold.

**Cluster:**	**Mes2**		**MyoF_T1**		**MyoF_P**	
**Cell types**	**Mean**	**Fold**	**Mean**	**Fold**	**Mean**	**Fold**
VSMC	0.12	2.49	0.18	5.11	0.11	6.86
HSC	**0.31**	1.00	0.32	2.91	0.25	3.09
MyoF	0.06	4.80	**0.92**	1.00	**0.78**	1.00
Meso	-0.07	-4.48	0.49	1.89	0.39	2.00

Table S7B. One-way ANOVA analysis for the four cell types in different clusters**.**

**Cluster**	**Source of Variation**	**Sum of Squares**	**Degrees of Freedom**	**Mean Square**	***F*-Calculated**	***P*-Value**
**Mes2**	Cell Types	28.54	3	9.51	371	$<2\text{x}10^{-16}$
	Residuals	59.03	2299	0.026		
**MyoF_T1**	Cell Types	137.27	3	45.76	2834	$<2\text{x}10^{-16}$
	Residuals	37.12	2299	0.02		
**MyoF_P**	Cell Types	110.5	3	36.83	3850	$<2\text{x}10^{-16}$
	Residuals	22	2299	0.01		

Table S7C. Tukey test for multiple comparisons of the module scores.

**Cluster**	**Pairwise Comparison**	**Mean Difference**	***P*-adjusted**	**95% Confidence Interval**	
				**Lower Bound**	**Upper Bound**
**Mes2**	HSC *vs* VSMC	0.19	$<1\times10^{-10}$	0.17	0.20
	MyoF *vs* HSC	-0.24	$<1\times10^{-10}$	-0.27	-0.22
	Meso *vs* HSC	-0.38	$<1\times10^{-10}$	-0.42	-0.34
**MyoF_T1**	MyoF *vs* VSMC	0.74	$<1\times10^{-10}$	0.72	0.76
	MyoF *vs* HSC	0.61	$<1\times10^{-10}$	0.59	0.62
	Meso *vs* MyoF	-0.44	$<1\times10^{-10}$	-0.47	-0.34
**MyoF_P**	HSC *vs* VSMC	0.67	$<1\times10^{-10}$	0.65	0.69
	MyoF *vs* HSC	0.53	$<1\times10^{-10}$	0.51	0.55
	Meso *vs* HSC	-0.39	$<1\times10^{-10}$	-0.41	-0.36

Table S8. Summary of the GSEA results assessing the correlation between the MyoF_T1 and MyoF_P gene signatures with NASH **(A**) or hepatocellular carcinoma-associated (HCC) **(B**) non-fibrotic and fibrotic liver tissues. Genes whose expression levels were increased in MyoF_T1 (n=527) or MyoF_P (n=1716) relative to Mes2 were used to form myofibroblast-specific gene expression signatures. The expression data was obtained from (A) early (non-fibrotic) stage 1 and late (fibrotic) stage 4 NASH liver tissue (GSE13525 (2)) or (B) from resected HCC liver tissue, which was classified by pathologists as fibrotic or non-fibrotic (GSE6764 (8)). GSEA analyses revealed that the MyoF_T1 signature was strongly associated with stage 4 NASH, but not with early stage 1 NASH liver tissue; and with liver fibrosis caused by HCC, but not with non-fibrotic HCC liver tissue. The MyoF_P signature had positive associations with stage 4 NASH and HCC-induced fibrotic liver tissue, but those associations were not statistically significant. The expression score (ES), normalized expression score (NES), nominal p-value, and false discovery rate (FDR q-value) are shown for each comparison.

A. NASH Analyses (GSE135251)

Comparison: Stage 4 (Fibrosis)

	ES	NES	P-value	FDR q-value
MyoF_T1	0.56	1.56	0.007	0.018
MyoF_P	0.45	1.32	0.10	0.15

Comparison: Stage 1 (No Fibrosis)

	ES	NES	P-value	FDR q-value
MyoF_T1	-0.30	-0.85	0.66	1
MyoF_P	-0.29	-0.82	0.72	0.87

B. HCC Analyses (GSE6764)

Comparison: Fibrotic Liver

	ES	NES	P-value	FDR q-value
MyoF_T1	0.51	1.71	0.0019	0.05
MyoF_P	0.36	1.48	0.03	0.06

Comparison: Non-fibrotic liver

	ES	NES	P-value	FDR q-value
MyoF_T1	-0.36	-1.32	0.10	0.16
MyoF_P	-0.34	-1.34	0.05	0.22

Table S9. The results of 2-way ANOVA analyses of the data shown in the indicated figures are shown. For these analyses, time and treatment group was treated as categorical variables. The degrees of freedom (DF), the sum of the squares (Sum Sq) and mean squared (mean Sq) of the variance, calculated F-value, and the probability (Pr(>F) from the 2-way ANOVA are shown for the treatment group, time, and treatment-by-time analyses. A Pr(>F) < 0.01 indicates that there was a significant effect of the indicated variable.

Figure 2F	DF	Sum Sq	Mean Sq	F value	Pr(>F)
Treatment	3	6.11E+14	2.04E+14	110.79	<2.00E-16
Time	4	1.47E+16	3.67E+14	19.98	4.92E-13
Treatment by Time	12	2.48E+14	2.07E+13	11.23	1.66E-15
Residuals	140	2.57E+14	1.84E+12		

Figure 7A	Df	Sum Sq	Mean Sq	F value	Pr(>F)
Treatment	6	2.96E+14	4.93E+13	14.087	1.24E-12
Time	2	1.08E+15	5.38E+14	153.846	<2.00E-16
Treatment by Time	12	1.44E+14	1.20E+13	3.431	0.000185
Residuals	147	5.14E+14	3.50E+12		

Figure 7B	Df	Sum Sq	Mean Sq	F value	Pr(>F)
Treatment	5	1.15E+14	2.30E+13	13.919	1.63E-10
Time	2	7.34E+14	3.67E+14	222.459	<2.00E-16
Treatment by Time	10	7.65E+13	7.65E+12	4.637	1.69E-05
Residuals	109	1.80E+14	1.65E+12		

Figure 6B	Df	Sum Sq	Mean Sq	F value	Pr(>F)
Treatment	3	1.22E+17	4.06E+16	75.096	<2.00E-16
Time	4	2.54E+16	6.36E+15	11.75	2.95E-08
Treatment by Time	12	5.57E+16	4.64E+15	8.578	4.56E-12
Residuals	140	7.57E+16	5.41E+14		

Figure 6C	Df	Sum Sq	Mean Sq	F value	Pr(>F)
Treatment	3	1.06E+15	3.52E+14	54.89	<2.00E-16
Time	2	5.33E+14	2.66E+14	41.51	1.02E-12
Treatment by Time	6	6.49E+14	1.08E+14	16.86	4.78E-12
Residuals	72	4.62E+14	6.42E+12		

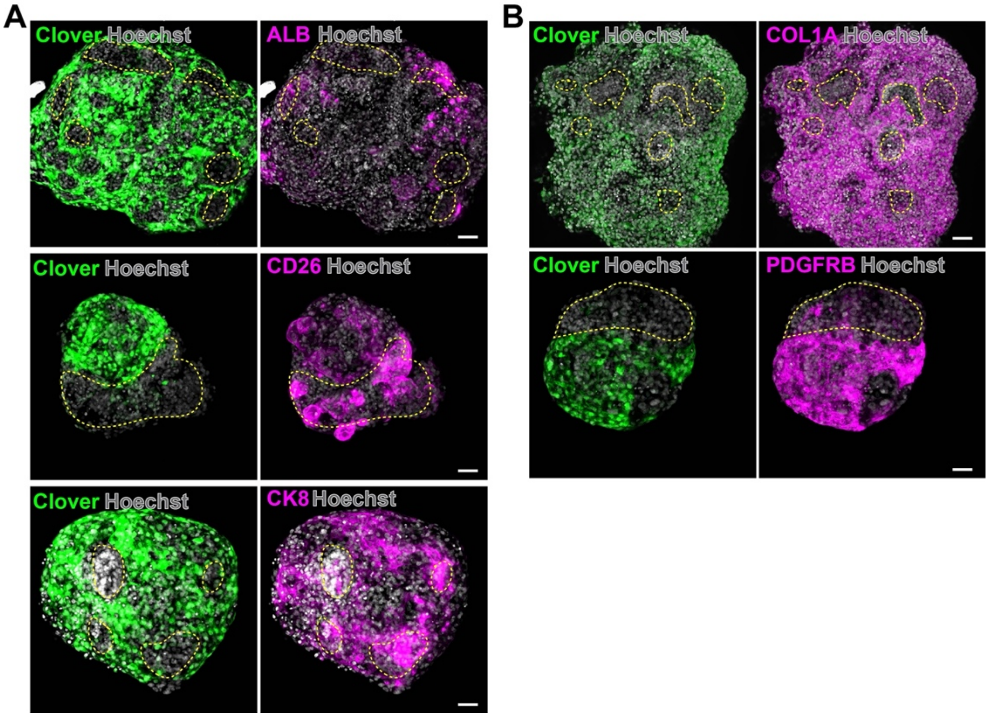

Figure S1. Immunofluorescent images of day 20 *COL1A1*-P2A Clover HOs stained with antibodies to Clover, Albumin (Alb), CD26 and CK8 (**A**); or antibodies to Clover, COL1A and PDGFRB (**B**). As seen in the merged images, the $Clover^{+}$ cells are distinct from the ALB^{+} and $CD26^{+}$ and/or $CK8^{+}$ hepatocytes and cholangiocytes; while PDGFRB and COL1A are co-expressed in the $Clover^{+}$ cells. The dotted lines surround areas that are not stained with Clover and the Hoechst staining identifies nuclei. Scale bars, 50 μm.

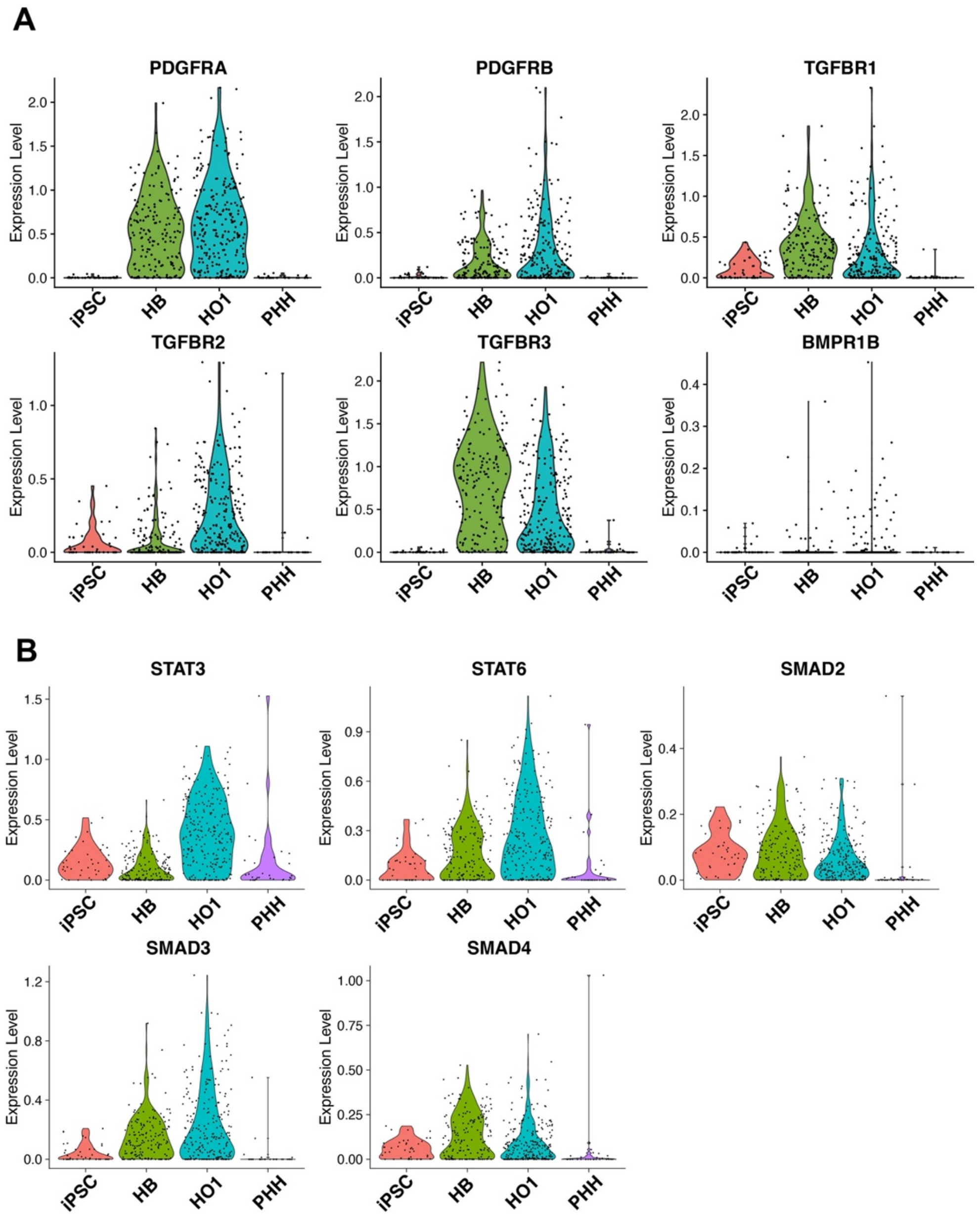

Figure S2. (**A, B**) Violin plots showing the level of expression of mRNAs encoding the receptors for PDGF (*PDGFRA, PDGFRB*) and TGFβ1 (*TGFBR1, TGFBR2, TGFBR3, BMPR1B*); and of *STAT3*, *STAT6*, and *SMAD2-4* mRNAs in developing and mature HO cultures. Previously obtained scRNA-Seq data (17) was generated from iPSC (day 0), day 9 hepatoblast (HB) and day 21 mature organoid cultures (HO1). For comparison purposes, scRNA-Seq data obtained from primary human hepatocytes (PHH) is also shown.

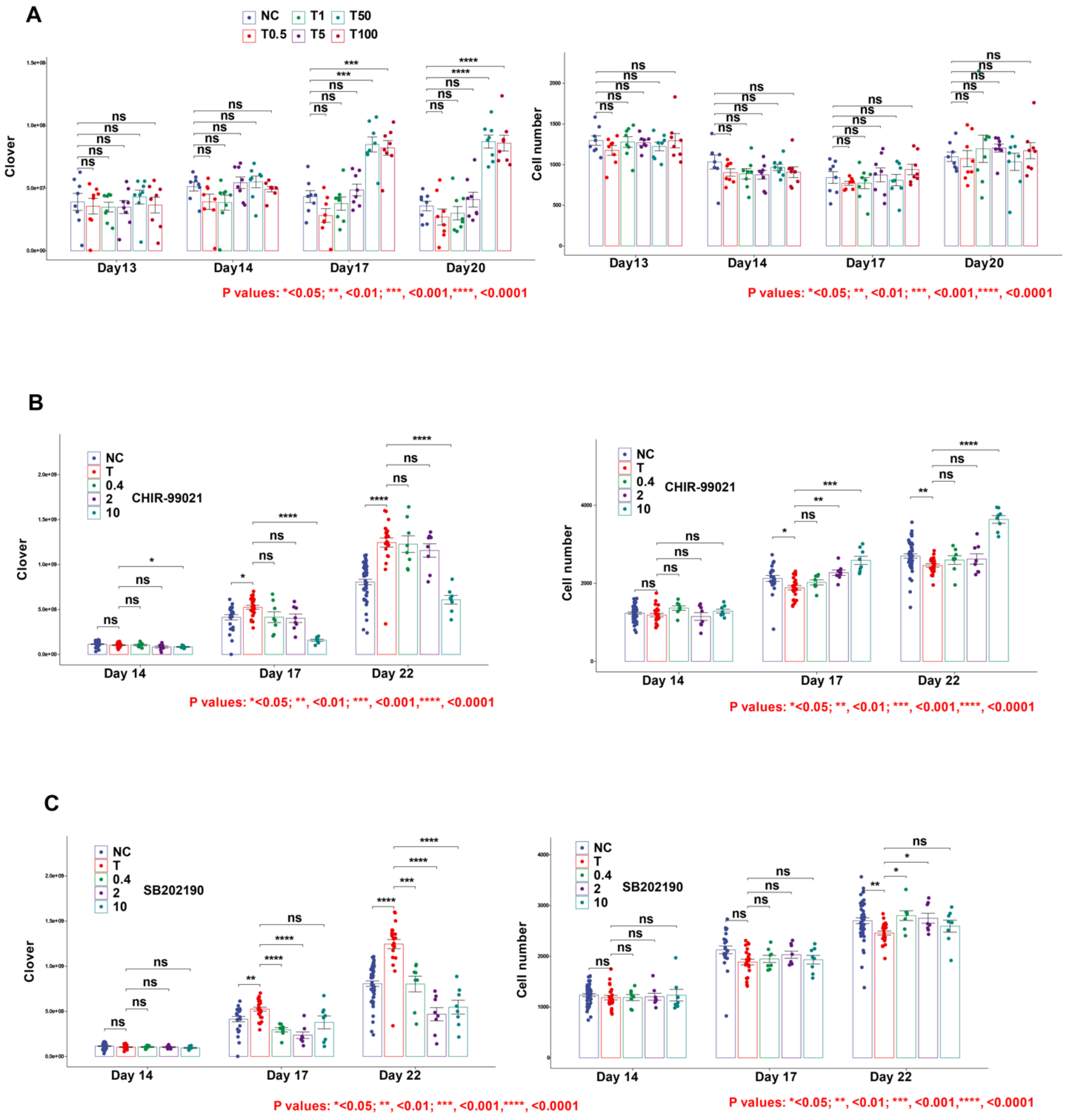

Figure S3. Titration of the effect of TGFB, and of GSK3β and p38 inhibitors on fibrosis and cell viability in microHOs. (**A**). TGFB Titration. (Left) Clover fluorescence (fibrosis) and cell number (right) induced by TGFB at concentrations ranging from 0 to 100 ng/ml administered on day 13. The measurements were obtained from day 13 to 20. The 50 ng/ml dose produces a maximal induction compared to normal control, but this did not alter cell viability. (**B**). GSK3β inhibitor (CHIR-99021) titration (Left) Clover fluorescence (fibrosis) induced by 50 ng/ml TGFB after treatment 0 .4 to 10 uM GSK3β inhibitor. A concentration of 2 uM or greater significantly inhibits TGFB-induced fibrosis in microHOs but had no effect on cell number (organoid viability). (**C**) p38 inhibitor (SB202190) titration. Clover fluorescence (fibrosis) induced by 50 ng/ml in the presence of 0.4 to 10 uM p38 inhibitor was significantly inhibited (left), while the cell number (organoid viability, right) was not affected by drug treatment.

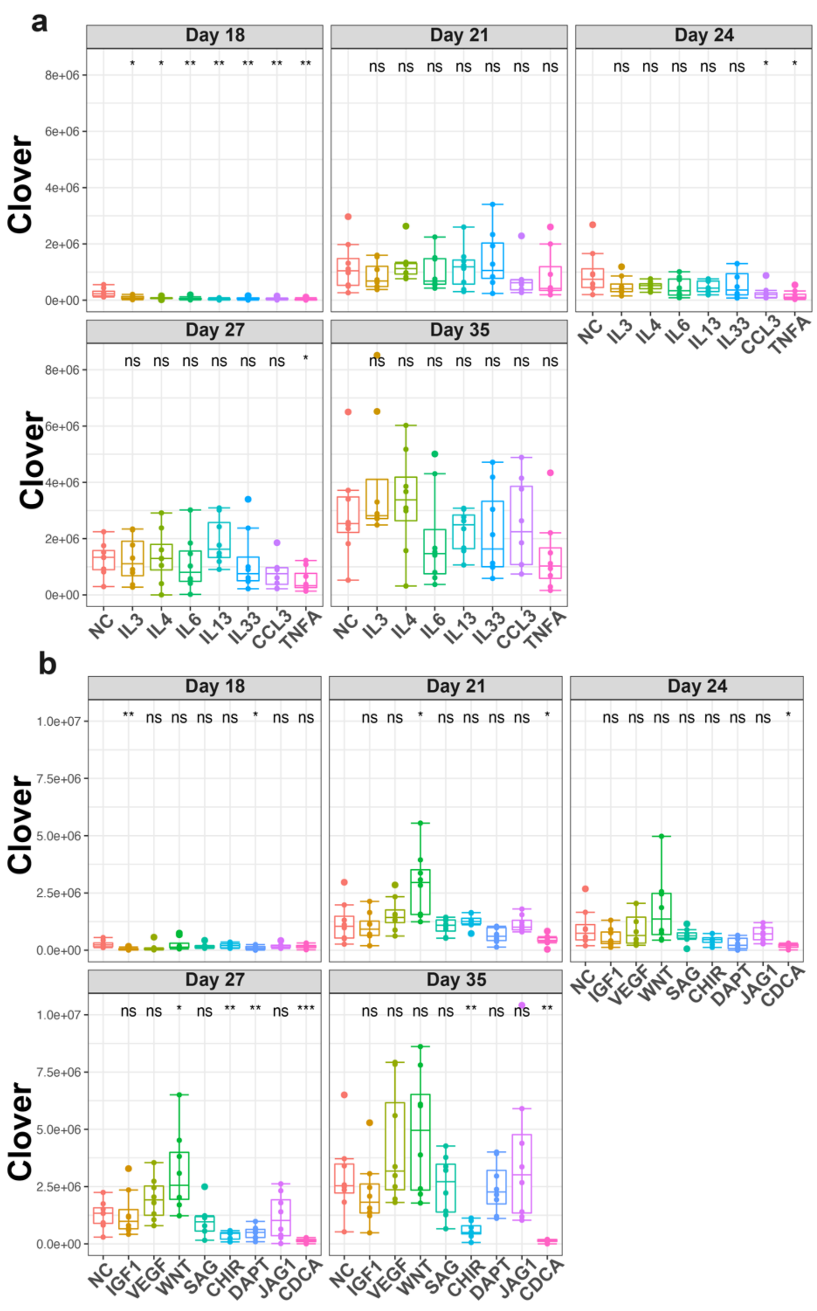

Figure S4. *COL1A1*-P2A Clover HOs were incubated with (**A**) 100 ng/ml IL-3, 100 ng/ml IL-4, 100 ng/ml IL-6, 100 ng/ml IL13, 100 ng/ml IL-33, 100 ng/ml CCL3, 50 ng/ml TNFa; or (**B**) 100 ng/ml IGF1, 50 ng/ml VEGF, 100 ng/ml Wnt3a, 10 μM SAG (Smoothened agonist), 3 μM CHIR99021, 10 μM DAPT (Gamma-Secretase Inhibitor), 100 ng/ml JAG1, 10 μM chenodeoxycholic acid (CDCA), or no addition (NC). Culture fluorescence (y-axis), which indicates the amount of COL1A1^{+} cells in the HOs, was serially measured on days 18 through

35. Each dot represents a measurement made on one HO, the thick line is the median of 8 organoids analyzed per condition, and the box plot shows the 25 to 75% range for all measurements per condition. With the possible exception of Wnt3a, none of these added agent caused a significant and or a sustained increase in $COL1A1^+$ cells in any of the organoid cultures. CDCA induced a decrease in $COL1A1^+$ cells.

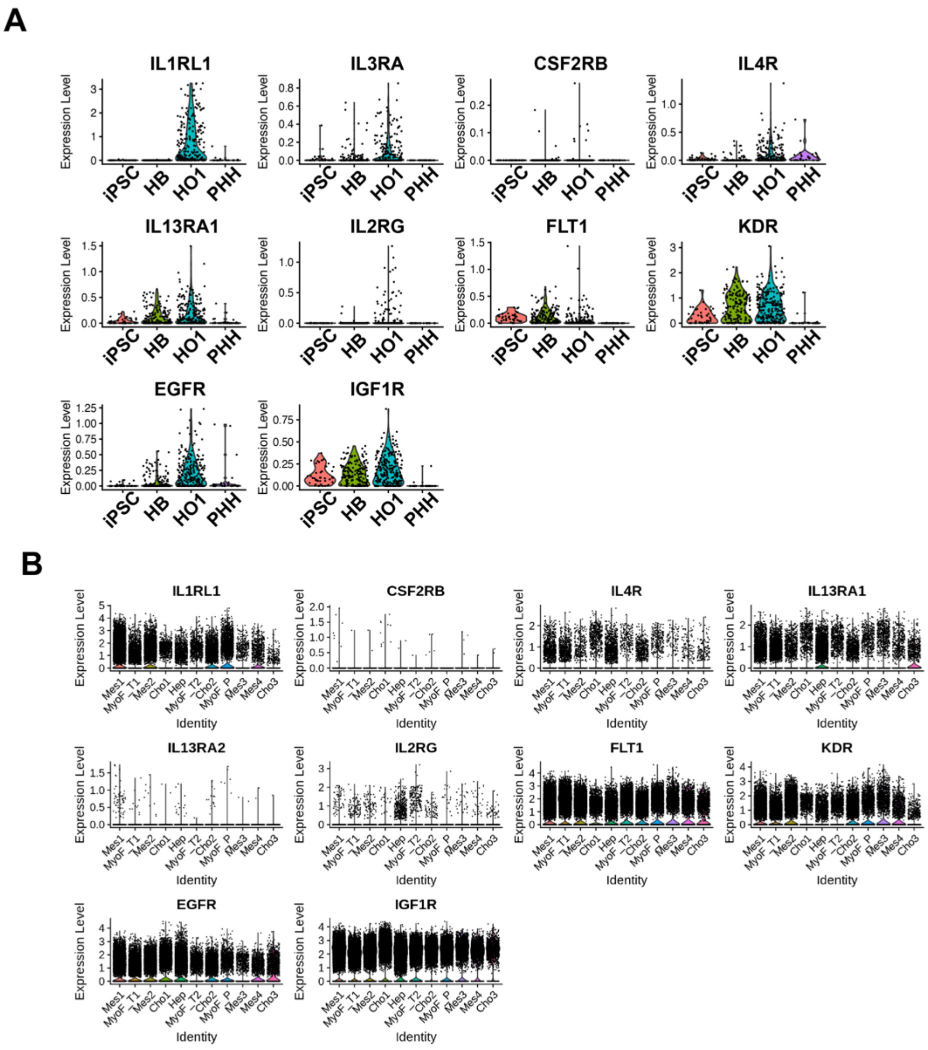

Figure S5. (**A**) Violin plots showing the level of expression of mRNAs for the following receptors during HO development: *IL1RL1, IL4R, IL13RA1, IL2RG, FLT1, KDR,* and *IGF1R*. scRNA-Seq data was generated from iPSC (day 0), day 9 hepatoblast (HB) and day 21 mature organoid (HO1) cultures. For comparison purposes, scRNA-Seq obtained from primary human hepatocytes (PHH) is also shown. This scRNA-Seq dataset was obtained from (17). (**B**) Violin plots showing the level of mRNA expression for the receptors shown in (A) in the 11 cell clusters identified in day 21 control, PDGF- and TGFβ-treated microHOs using the scRNA-Seq data generated in this paper.

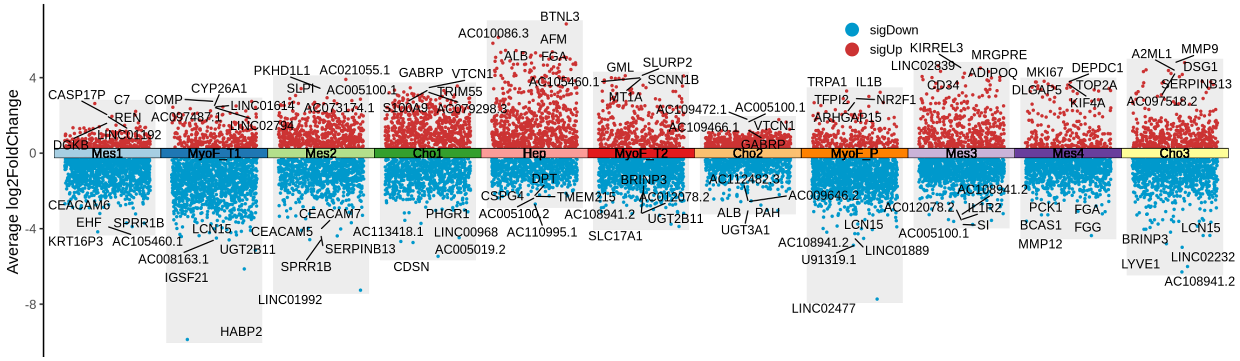

Figure S6. A combination volcano plot showing the top 5 DEGs for each of the 11 clusters. The red (or blue) dots indicate up-regulated (or down-regulated genes. As examples, Mes4 is the most proliferative cluster, and *MKI67* and *TOP2A* were among its DEGs; and the DEGs for the Hep cluster included hepatocyte specific markers (*ALB* and *FGA*).

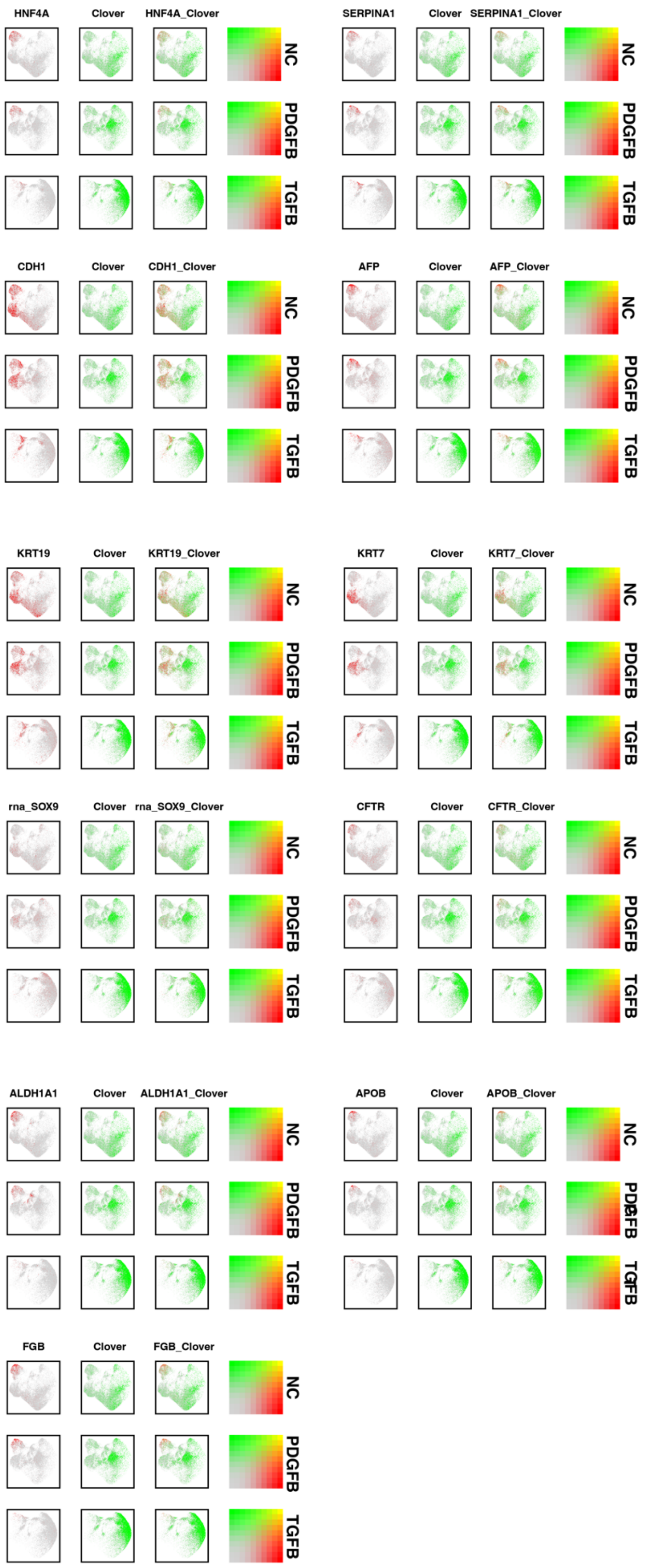
HNF4A
Clover
HNF4A_Clover
NC
PDGFB
TGFB
SERPINA1
Clover
SERPINA1_Clover
NC
PDGFB
TGFB
CDH1
Clover
CDH1_Clover
NC
PDGFB
TGFB
AFP
Clover
AFP_Clover
NC
PDGFB
TGFB
KRT19
Clover
KRT19_Clover
NC
PDGFB
TGFB
KRT7
Clover
KRT7_Clover
NC
PDGFB
TGFB
rna_SOX9
Clover
rna_SOX9_Clover
NC
PDGFB
TGFB
CFTR
Clover
CFTR_Clover
NC
PDGFB
TGFB
ALDH1A1
Clover
ALDH1A1_Clover
NC
PDGFB
TGFB
APOB
Clover
APOB_Clover
NC
PDGFB
TGFB
FGB
Clover
FGB_Clover
NC
PDGFB
TGFB

Figure S7. Feature plots show the level of expression of each indicated mRNA with *Clover* mRNA in the u-MAP plots shown in Fig 3. The uMAP plots for the normal control (NC), PDGF-, and TGFβ-treated microHOs are shown separately. The colors represent level of expression of the two mRNAs in each row as shown in the adjacent color threshold diagram. The mRNAs found in cholangiocytes (*KRT19*, *KRT7*, *CFTR),* hepatocytes *(SERPIN1A)* or hepatocytes and cholangiocytes (*HNF4A, AFP, CDH1, ALDHA1, APOB, SOX9, FGB*) were not expressed in the cells that expressed *Clover* mRNA in the microHOs.

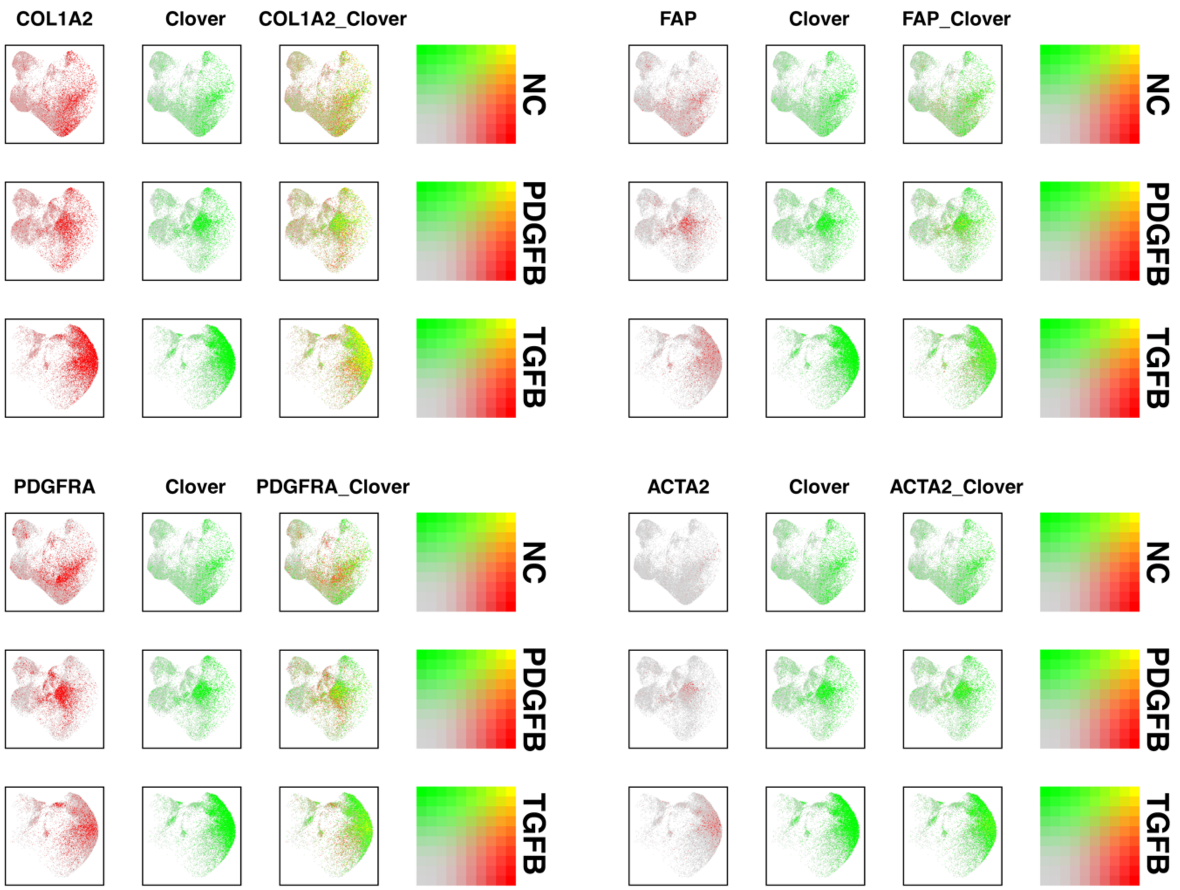

Figure S8. Feature plots show the level of expression of *COL1A2, PDGFRA, FAP or ACTA2* mRNAs with *Clover* mRNA in the u-MAP plots shown in Fig 3A. The UMAP plots for the normal control (NC), PDGF-, and TGFβ-treated microHOs are shown separately. The colors represent level of expression of the two mRNAs in each row as shown in the adjacent color threshold diagram. *COL1A2 and Clover* mRNAs have an overlapping pattern of expression; they are expressed in myofibroblasts and in mesenchymal cells. In contrast, *ACTA2, PDGFRA* and *FAP* mRNAs are predominantly expressed in the myofibroblasts (MyoF-T1/2, MyoF_P) in PDGF- or TGFβ-treated microHOs.

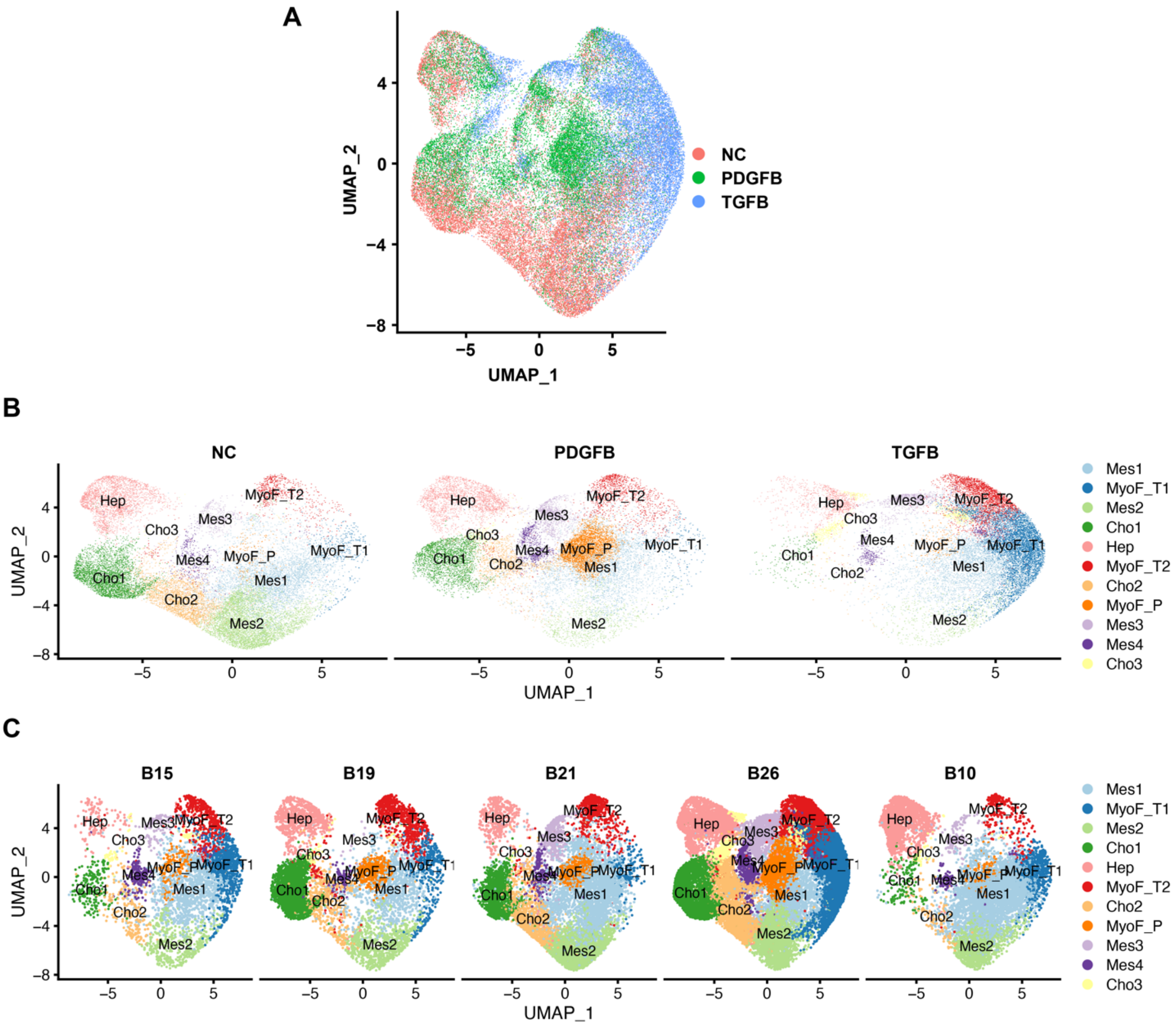

Figure S9. (**A**) A combined sample u-MAP plot shows the scRNA-seq data obtained from the cells in day 21 control (NC), PDGFβ-, or TGFβ1-treated microHO cultures. Each of the different type of microHO is indicated by the color shown on the right of the panel. (**B**) Separate u-MAP plots show the scRNA-seq data obtained from the cells in day 21 control (NC), PDGFβ-, or TGFβ1-treated microHO cultures. (**C**) The u-MAP plots for each of the five batches of microHOs that were separately analyzed by scRNA-Seq analysis. Each cluster is indicated by a different color as indicated on the right side of panels B and C.

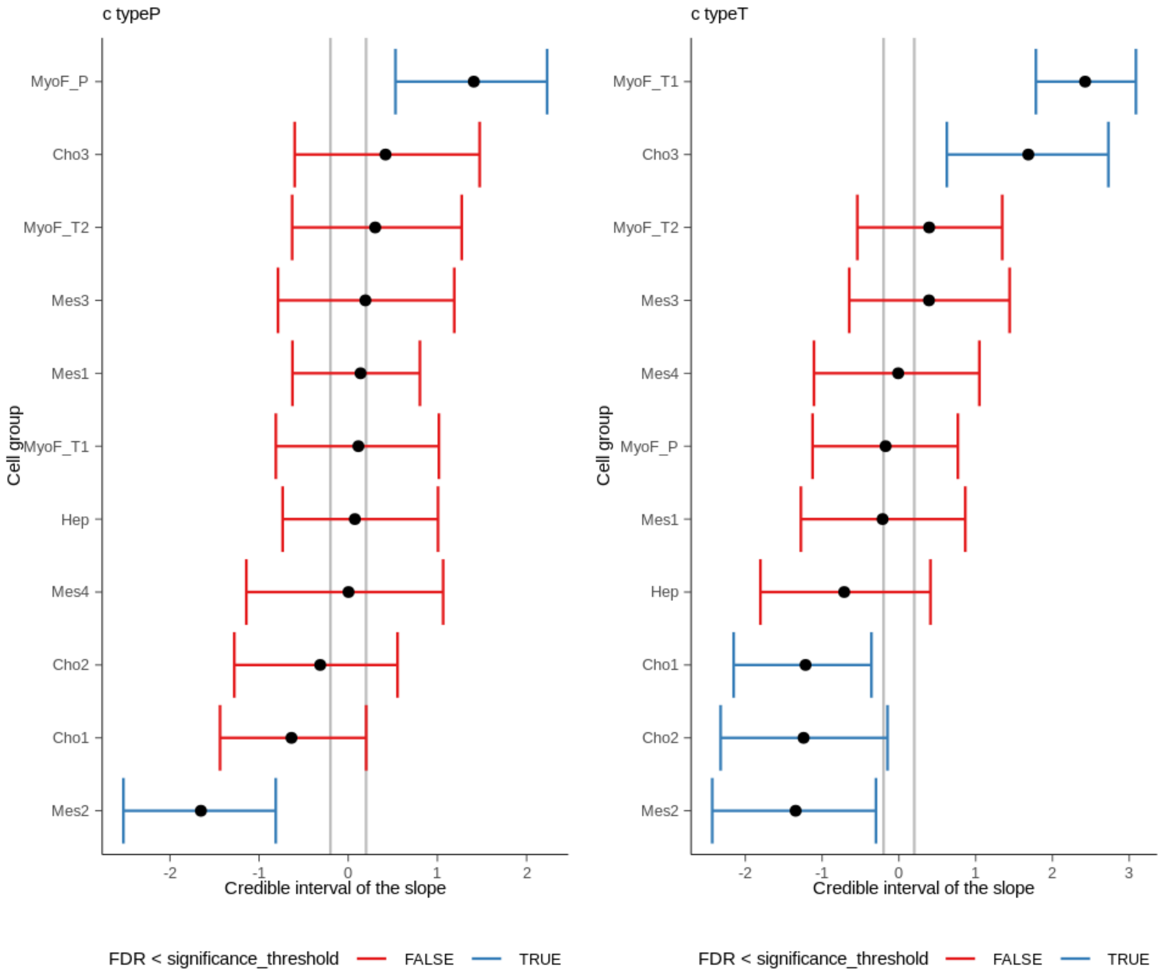

Figure S10. (**A**) Plots show the credible intervals determined by sccomp for each cell cluster in PDGFB-treated (Left) or TGFβ-treated (Right) microHOs vs NC microHOs. A blue color indicates that the treatment caused a significant change in the cell percentage.

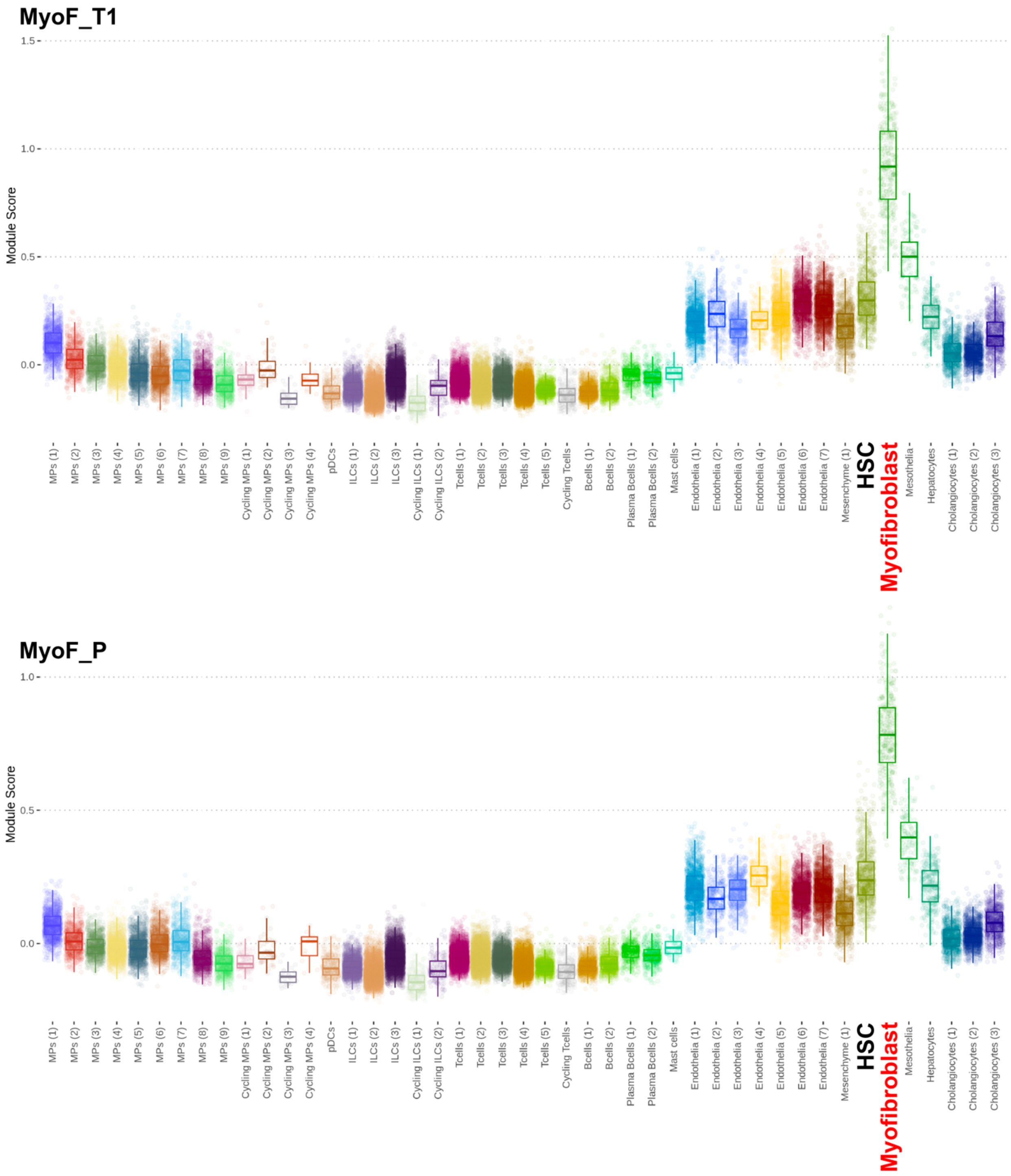

Figure S11. Among the multiple different types of cells present in normal and cirrhotic human liver, the MyoF_T1 and MyoF_P transcriptomes are most like that of myofibroblasts. The MyoF_T1 (n=53 genes) or MyoF_P (n=84 genes) signatures were compared with the transcriptomes of the various cell types in normal and cirrhotic human liver tissue (GSE136103 (18)), which include macrophages (MP) and innate lymphoid cells (ILC).). Each dot shows the modulus score obtained when the gene signatures for the cell type indicated on the x-axis was compared with that of MyoF_T1 or MyoF_P. The thick line shows the mean, the boxplots show the 25 to 75% range, and the vertical line shows the Minimum (Q0 or 0th percentile) and

Maximum (Q4 or 100th percentile) of the scores. The MyoF_T1 and MyoF_P signatures are most enriched in the myofibroblasts in human liver tissue. The dotted lines represent actual reference module scores.

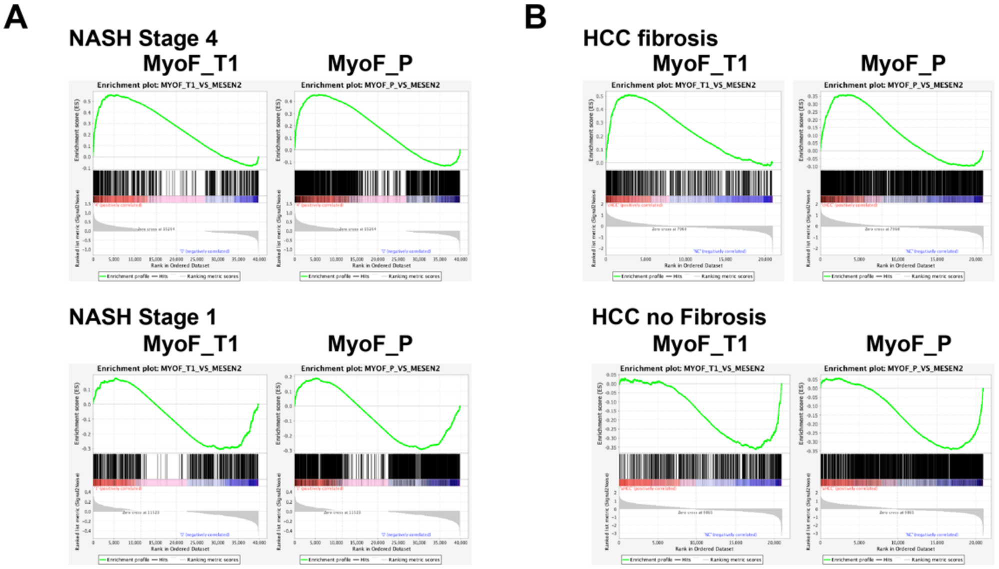

Figure S12. GSEA results assessing the correlation between the MyoF_T1 and MyoF_P gene signatures with that of non-fibrotic and fibrotic liver tissues caused by NASH **(A**) or hepatocellular carcinoma (HCC) **(B**). Genes whose expression levels were increased in MyoF_T1 (n=527) or MyoF_P (n=1716) relative to Mes2 (**Table S*)** were used to form myofibroblast-specific gene expression signatures. GSEA was performed using expression data obtained from (A) early (non-fibrotic) stage 1 and late (fibrotic) stage 4 NASH liver tissue (GSE13525 (2)) or (B) from resected HCC liver tissue, which was classified by histologic analysis as fibrotic or non-fibrotic (GSE6764 (8)). GSEA analyses revealed that the MyoF_T1 (NSE 1.7; false discovery rate (FDR) $3.3x10^{-4}$) and MyoF_P (1.4; FDR 0.013) signatures were strongly associated with stage 4 NASH liver tissue, but not with early (NES -0.97 and -0.95, FDR 1 and 0.99, respectively) stage 1 NASH liver tissue; and the MyoF_T1 (NES 2.77, FDR 0) and MyoF_P (NES 1.8, FDR $4.4x10^{-4}$) signatures were associated with liver fibrosis caused by HCC, whereas the MyoF_T1 (NES -1.98, FDR 0) and MyoF_P (NES -1.97, FDR 0) signatures were not associated with non-fibrotic HCC liver tissue.

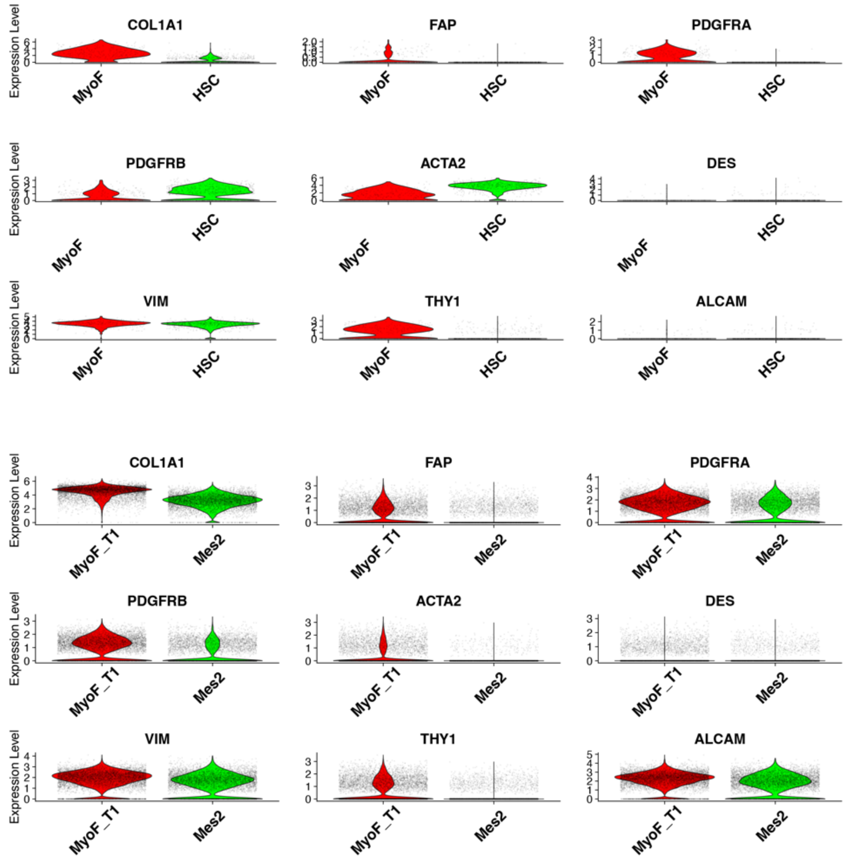

Figure S13. The pattern of expression of mesenchyme and MyoF-specific genes in hepatic stellate cells (HSC) and MyoF in human liver is mostly retained in Mes2 and MyoF_T1 cells in microHOs. scRNA-Seq data was analyzed to generate violin plots showing the level of expression of *COL1A1, FAP, PDGFRA, PDGFRB, ACTA2, DES, VIM, Thy1* and *ALCAM* mRNAs in HSC and MyoF in cirrhotic human liver (GSE136103; Top panel) and in Mes2 and MyoF_T1 cells in microHOs (Bottom panel). The cell cluster is indicated on the x-axis; and the y-axis shows the natural log transformed and normalized level of expression of each mRNA. Each dot shows the expression level in one cell.

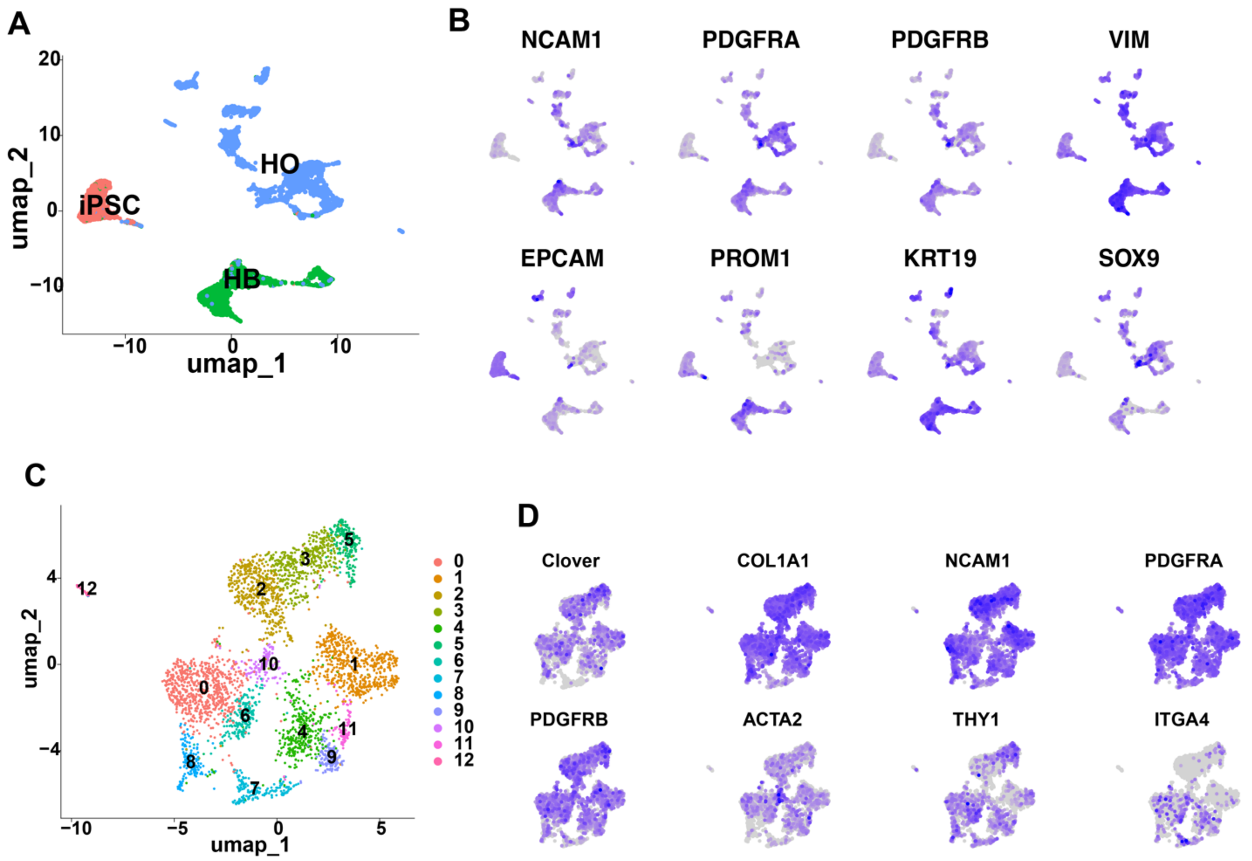

Figure S14. scRNA-seq reveal cell type heterogeneity in day 9 hepatoblast cultures. (**A**) UMAP of scRNA-Seq data obtained from iPSC, day 9 hepatoblasts and mature day 21HO cultures. (**B**) Feature plots show the level of expression of mesenchymal cell (NCAM1, PDGFRA, PDGFRB and VIM) and epithelial cell (EPCAM, PROM1, KRT19 and SOX9) mRNAs. (C) UMAP plots showing 13 clusters within hepatoblast cultures generated from analysis of scRNA-seq data. (D) These feature plots show the level of expression of Clover, COL1A1 and other mesenchymal cell marker (NCAM1, PDGFRA, PDGFRB, ACTA2, THY1 and ITGA4) mRNAs in hepatoblast cultures.

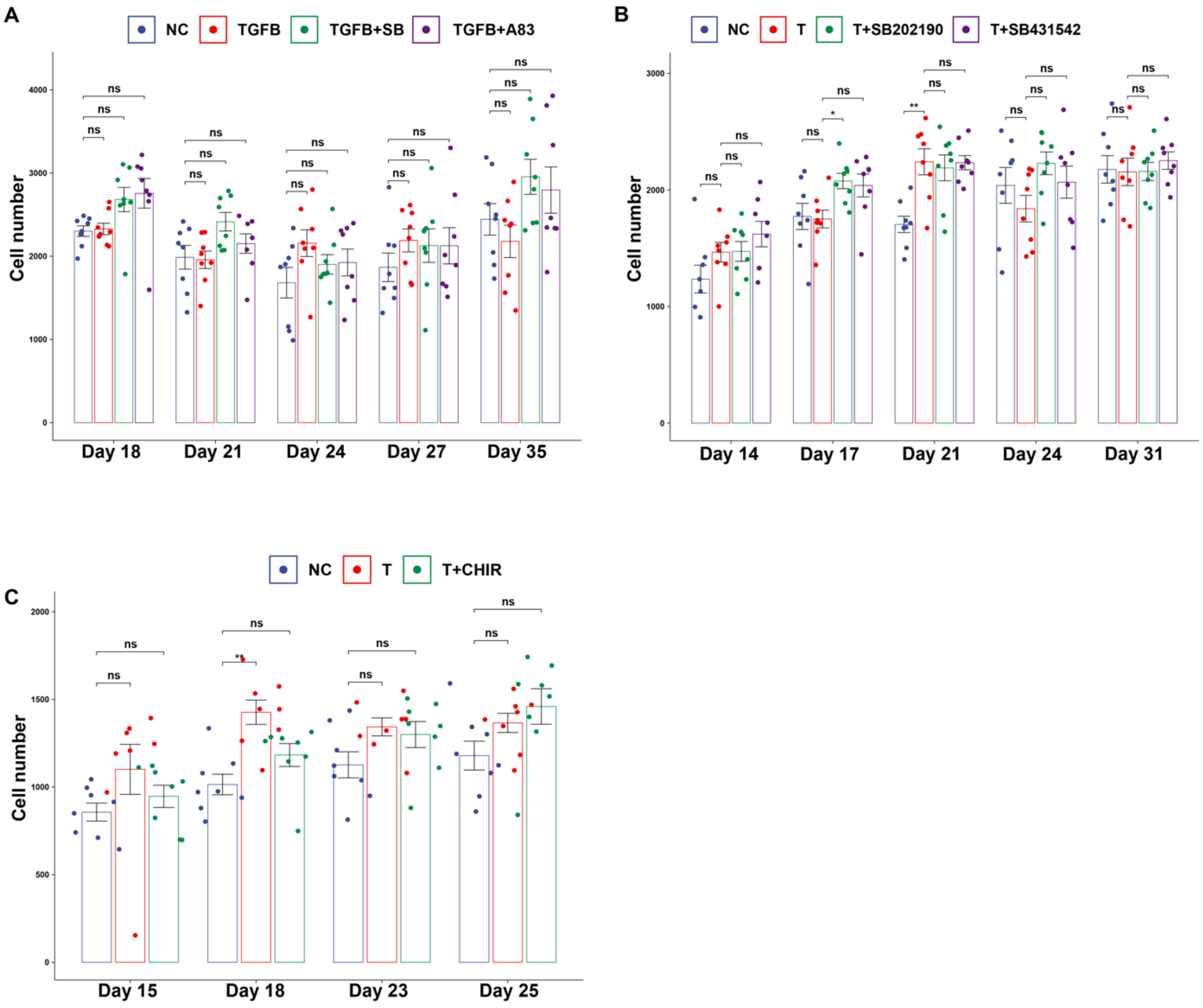

Figure S15. microHO viability was not affected by addition of TGFβ1, nor by any of the other tested drugs. microHOs were prepared and incubated with no addition (NC), 50 ng/ml TGFβ1 (T), or TGFβ1 ± the indicated inhibitor on day 13. The tested drugs were: (**A)** 10 uM TGFRβ1 tyrosine kinase inhibitors A83-01 or SB431542 (SB); (**B**) 10 uM TGFRβ1 inhibitor (SB431542) or 10 uM p38 α inhibitor (SB202190); and (**C**) 10 uM CHIR99021 (CHIR). The number of cells in each microHO was measured by counting the number of nuclei (stained with Hoechst 33342) on days 14 to 35. Each bar is the average of 8 individual measurements ± the SE, and individual datapoints are shown as dots. None of these additions caused a significant change (ns) in the number of cells in the treated microHOs relative to control microHOs.

Table S4. Twenty mRNAs that are differentially expressed in each of the 11 cell clusters identified in microHOs. The gene symbol, p-value, adjusted p-value, and fold differential expression (log_2) for each differentially expressed gene are shown. Pct.1 and Pct.2 indicates the contribution of that feature to defining the indicated cluster based upon principal component analysis.

	cluster	gene	p_val	avg_log2FC	pct.1	pct.2	p_val_adj
**1**	0	*COL14A1*	0.00	0.74	0.77	0.55	0
**2**	0	*ADAMTS1*	0.00	0.69	0.87	0.6	0
**3**	0	*KCND2*	0.00	0.65	0.75	0.53	1.41073166685289E-295
**4**	0	*DLK1*	0.00	0.64	0.97	0.88	0
**5**	0	*IL1RL1*	0.00	0.63	0.6	0.4	6.07272463504603E-113
**6**	0	*COL6A3*	0.00	0.58	0.99	0.93	0
**7**	0	*IL6ST*	0.00	0.57	0.99	0.95	0
**8**	0	*ITGA1*	0.00	0.56	0.93	0.74	0
**9**	0	*ZNF804A*	0.00	0.55	0.85	0.66	9.89305658045823E-202
**10**	0	*CLMP*	0.00	0.55	0.92	0.68	0
**11**	0	*PDGFRA*	0.00	0.54	0.91	0.76	0
**12**	0	*SOX5*	0.00	0.54	1	0.99	0
**13**	0	*DCN*	0.00	0.54	0.98	0.89	0
**14**	0	*LAMA2*	0.00	0.53	0.96	0.8	0
**15**	0	*LUM*	0.00	0.51	0.96	0.82	0
**16**	0	*SERPINE2*	0.00	0.49	0.99	0.93	0
**17**	0	*DPP4*	0.00	0.49	0.97	0.85	0
**18**	0	*COL1A2*	0.00	0.48	1	0.99	2.39332526066761E-224
**19**	0	*RSPO2*	0.00	0.47	0.9	0.69	0
**20**	0	*TGFBR3*	0.00	0.45	0.88	0.65	0
**21**	1	*CILP*	0.00	2.11	0.98	0.8	0
**22**	1	*TIMP3*	0.00	1.99	1	0.96	0
**23**	1	*COMP*	0.00	1.96	0.87	0.67	0
**24**	1	*ITGA11*	0.00	1.52	0.88	0.64	0
**25**	1	*COL1A1*	0.00	1.49	1	0.98	0

**26**	1	*AC097487.1*	0.00	1.48	0.65	0.59	0
**27**	1	*TRABD2B*	0.00	1.43	0.9	0.64	0
**28**	1	*MXRA5*	0.00	1.37	0.96	0.79	0
**29**	1	*TIMP1*	0.00	1.32	1	0.97	0
**30**	1	*CCN2*	0.00	1.32	0.86	0.67	0
**31**	1	*ABTB2*	0.00	1.32	0.98	0.76	0
**32**	1	*LTBP2*	0.00	1.30	0.91	0.67	0
**33**	1	*NOX4*	0.00	1.27	0.93	0.76	0
**34**	1	*EGFP*	0.00	1.27	0.96	0.82	0
**35**	1	*SMYD3*	0.00	1.27	0.99	0.93	0
**36**	1	*AF165147.1*	0.00	1.23	0.81	0.71	0
**37**	1	*SPON1*	0.00	1.22	0.75	0.55	0
**38**	1	*SERPINE2*	0.00	1.22	1	0.94	0
**39**	1	*MICAL2*	0.00	1.17	0.96	0.79	0
**40**	1	*CLU*	0.00	1.17	0.92	0.73	0
**41**	2	*CNTN5*	0.00	2.15	0.88	0.64	0
**42**	2	*LINC01088*	0.00	2.05	0.97	0.71	0
**43**	2	*LINC02388*	0.00	2.05	0.93	0.66	0
**44**	2	*POSTN*	0.00	1.70	0.95	0.74	0
**45**	2	*NPNT*	0.00	1.67	0.92	0.73	0
**46**	2	*ARHGAP6*	0.00	1.63	0.88	0.59	0
**47**	2	*SLIT3*	0.00	1.58	0.91	0.78	0
**48**	2	*DIRC3*	0.00	1.48	0.91	0.68	0
**49**	2	*PKHD1L1*	0.00	1.44	0.76	0.51	0
**50**	2	*CLSTN2*	0.00	1.35	0.82	0.62	0
**51**	2	*BCO2*	0.00	1.35	0.94	0.69	0
**52**	2	*PDPN*	0.00	1.29	0.98	0.87	0
**53**	2	*CPS1*	0.00	1.28	0.94	0.7	0
**54**	2	*KCNMA1*	0.00	1.26	0.94	0.8	0
**55**	2	*LINC00894*	0.00	1.26	0.92	0.68	0
**56**	2	*PWWP3B*	0.00	1.25	0.87	0.59	0

**57**	2	*COL11A1*	0.00	1.25	0.98	0.87	0
**58**	2	*KDR*	0.00	1.24	0.87	0.59	0
**59**	2	*PRLR*	0.00	1.24	0.96	0.77	0
**60**	2	*GHR*	0.00	1.23	0.88	0.68	0
**61**	3	*UNC5C*	0.00	2.45	0.96	0.62	0
**62**	3	*GRHL2*	0.00	2.36	0.91	0.64	0
**63**	3	*ITGB6*	0.00	2.32	0.96	0.7	0
**64**	3	*GABRP*	0.00	2.29	0.86	0.58	0
**65**	3	*PDE4D*	0.00	2.20	0.96	0.81	0
**66**	3	*ARHGAP29*	0.00	2.11	0.89	0.51	0
**67**	3	*PLD5*	0.00	2.08	0.86	0.67	0
**68**	3	*ROR1*	0.00	2.08	0.86	0.56	0
**69**	3	*AC015522.1*	0.00	2.00	0.98	0.77	0
**70**	3	*PRTG*	0.00	1.93	0.83	0.51	0
**71**	3	*NRXN3*	0.00	1.88	0.89	0.66	0
**72**	3	*ST6GALNAC3*	0.00	1.85	0.89	0.66	0
**73**	3	*NEBL*	0.00	1.85	0.91	0.75	0
**74**	3	*ANKS1A*	0.00	1.81	0.88	0.77	0
**75**	3	*EYA1*	0.00	1.76	0.67	0.53	0
**76**	3	*NAALADL2*	0.00	1.75	0.84	0.65	0
**77**	3	*SEMA5A*	0.00	1.74	0.95	0.73	0
**78**	3	*PTN*	0.00	1.67	0.8	0.55	0
**79**	3	*THSD4*	0.00	1.65	0.84	0.56	0
**80**	3	*PATJ*	0.00	1.64	0.82	0.63	0
**81**	4	*AFP*	0.00	3.58	0.72	0.53	0
**82**	4	*CHST9*	0.00	1.90	0.77	0.39	0
**83**	4	*APOB*	0.00	1.82	0.51	0.46	1.57013013564819E-97
**84**	4	*CEACAM6*	0.00	1.79	0.65	0.44	0
**85**	4	*FGB*	0.00	1.71	0.55	0.45	7.57761739439617E-259
**86**	4	*LINC00511*	0.00	1.68	0.87	0.54	0
**87**	4	*NTN4*	0.00	1.68	0.71	0.55	0

**88**	4	*SERPINA1*	0.00	1.58	0.53	0.43	3.26816794412982E-195
**89**	4	*DNAJC15*	0.00	1.47	0.91	0.73	0
**90**	4	*CDH17*	0.00	1.44	0.58	0.44	0
**91**	4	*CDH2*	0.00	1.38	0.95	0.79	0
**92**	4	*FOXP2*	0.00	1.37	0.73	0.55	0
**93**	4	*EPCAM*	0.00	1.26	0.75	0.52	0
**94**	4	*APOA1*	0.00	1.25	0.62	0.37	0
**95**	4	*MARCHF1*	0.00	1.23	0.64	0.44	0
**96**	4	*CDH1*	0.00	1.23	0.77	0.55	0
**97**	4	*CCSER1*	0.00	1.21	0.8	0.54	0
**98**	4	*MAML3*	0.00	1.20	0.9	0.75	0
**99**	4	*LINC00278*	0.00	1.20	0.78	0.59	0
**100**	4	*LCP1*	0.00	1.16	0.76	0.55	0
**101**	5	*PI16*	0.00	2.48	0.92	0.74	0
**102**	5	*C3*	0.00	2.00	0.74	0.59	0
**103**	5	*IGFBP6*	0.00	1.93	0.81	0.67	0
**104**	5	*HTRA3*	0.00	1.86	0.77	0.63	0
**105**	5	*GABRB3*	0.00	1.69	0.4	0.47	0.645287107
**106**	5	*SLC24A2*	0.00	1.49	0.75	0.65	0
**107**	5	*KRT14*	0.00	1.32	0.65	0.58	0
**108**	5	*PRSS23*	0.00	1.30	0.83	0.69	0
**109**	5	*TIMP3*	0.00	1.21	1	0.96	0
**110**	5	*ATP10A*	0.00	1.19	0.78	0.69	0
**111**	5	*ANK2*	0.00	1.19	0.85	0.73	0
**112**	5	*TNS1*	0.00	1.17	0.87	0.73	0
**113**	5	*CSPG4*	0.00	1.16	0.7	0.59	0
**114**	5	*CD55*	0.00	1.14	0.72	0.67	4.49192035504069E-284
**115**	5	*KLF3*	0.00	1.10	0.78	0.71	0
**116**	5	*CPAMD8*	0.00	1.07	0.58	0.42	0
**117**	5	*AC099520.1*	0.00	1.05	0.5	0.5	2.92243869181999E-49
**118**	5	*ABI3BP*	0.00	1.04	0.76	0.67	0

**119**	5	*ZBTB7C*	0.00	1.03	0.57	0.58	3.96295910582397E-99
**120**	5	*FLNC*	0.00	1.02	0.73	0.61	0
**121**	6	*UNC5C*	0.00	0.84	0.94	0.63	0
**122**	6	*GABRP*	0.00	0.78	0.79	0.6	0
**123**	6	*ST6GALNAC3*	0.00	0.78	0.9	0.67	0
**124**	6	*GRHL2*	0.00	0.77	0.89	0.65	0
**125**	6	*PRTG*	0.00	0.71	0.82	0.53	0
**126**	6	*PDE4D*	0.00	0.69	0.96	0.82	0
**127**	6	*ROR1*	0.00	0.69	0.85	0.57	0
**128**	6	*ARHGAP29*	0.00	0.68	0.83	0.54	0
**129**	6	*ANKS1A*	0.00	0.65	0.91	0.77	0
**130**	6	*ITGB6*	0.00	0.65	0.93	0.71	0
**131**	6	*PLD5*	0.00	0.62	0.84	0.68	0
**132**	6	*LINC02388*	0.00	0.61	0.74	0.69	3.14703394653217E-146
**133**	6	*EYA1*	0.00	0.60	0.67	0.54	1.6275168304562E-266
**134**	6	*DIAPH3*	0.00	0.60	0.68	0.55	1.51594497149573E-217
**135**	6	*NRXN3*	0.00	0.59	0.88	0.67	0
**136**	6	*FREM2*	0.00	0.59	0.66	0.52	1.11716953189223E-242
**137**	6	*KCNQ5*	0.00	0.58	0.73	0.57	5.17999574470969E-237
**138**	6	*AC015522.1*	0.00	0.58	0.97	0.78	0
**139**	6	*PTN*	0.00	0.57	0.71	0.57	6.4862311734237E-293
**140**	6	*SEMA5A*	0.00	0.57	0.94	0.74	0
**141**	7	*TFPI2*	0.00	2.00	0.62	0.46	0
**142**	7	*PAPPA*	0.00	1.65	0.98	0.89	0
**143**	7	*PDE5A*	0.00	1.55	0.73	0.57	0
**144**	7	*ADAMTS1*	0.00	1.53	0.8	0.65	0
**145**	7	*AREG*	0.00	1.35	0.69	0.64	5.25882782978605E-270
**146**	7	*ITGA1*	0.00	1.26	0.88	0.77	0
**147**	7	*ABCC4*	0.00	1.23	0.71	0.64	0
**148**	7	*MSC-AS1*	0.00	1.20	0.55	0.51	1.60206265130991E-185
**149**	7	*PIEZO2*	0.00	1.13	0.62	0.48	3.62254977687614E-291

**150**	7	*SUCLG2-AS1*	0.00	1.12	0.73	0.65	0
**151**	7	*CLMP*	0.00	1.11	0.79	0.72	0
**152**	7	*KYNU*	0.00	1.07	0.45	0.5	1.09972579275846E-41
**153**	7	*IGFBP5*	0.00	1.06	0.86	0.8	0
**154**	7	*ROBO2*	0.00	1.05	0.84	0.71	0
**155**	7	*RORB*	0.00	1.05	0.64	0.65	1.71419981781713E-181
**156**	7	*MASP1*	0.00	0.98	0.6	0.52	2.14815931343417E-221
**157**	7	*AL691420.1*	0.00	0.94	0.69	0.74	1.90329965031377E-129
**158**	7	*KCND2*	0.00	0.94	0.6	0.58	1.12904013826135E-87
**159**	7	*PAMR1*	0.00	0.93	0.66	0.64	1.05279467805712E-135
**160**	7	*MMP16*	0.00	0.91	0.79	0.73	6.45407093018707E-251
**161**	8	*CXCL14*	0.00	2.54	0.82	0.61	0
**162**	8	*KIRREL3*	0.00	2.47	0.64	0.58	1.78087718527724E-275
**163**	8	*NRP2*	0.00	2.07	0.81	0.59	0
**164**	8	*GSN*	0.00	2.01	0.93	0.84	0
**165**	8	*AC002463.1*	0.00	1.95	0.66	0.56	0
**166**	8	*CRHBP*	0.00	1.89	0.53	0.52	2.68844774508409E-87
**167**	8	*ATRNL1*	0.00	1.84	0.67	0.58	7.4046528162926E-205
**168**	8	*NRG3*	0.00	1.81	0.78	0.51	0
**169**	8	*CDH13*	0.00	1.79	0.63	0.48	1.90104856701329E-243
**170**	8	*MIAT*	0.00	1.79	0.7	0.56	0
**171**	8	*NTM*	0.00	1.77	0.59	0.55	1.11151682293596E-121
**172**	8	*PLCB4*	0.00	1.76	0.74	0.59	0
**173**	8	*OXCT1*	0.00	1.76	0.61	0.56	1.22313326537527E-195
**174**	8	*NPAS2*	0.00	1.59	0.67	0.53	1.64759745644261E-255
**175**	8	*PI16*	0.00	1.56	0.8	0.76	2.48410109644837E-303
**176**	8	*CD109*	0.00	1.54	0.75	0.7	3.32247596107369E-263
**177**	8	*LINC01592*	0.00	1.53	0.54	0.55	4.53240260414655E-120
**178**	8	*TEK*	0.00	1.48	0.73	0.63	7.56466873318405E-289
**179**	8	*PARD3B*	0.00	1.45	0.76	0.7	1.08312332593575E-226
**180**	8	*PODXL*	0.00	1.43	0.76	0.7	2.30063348380999E-244

**181**	9	*MKI67*	0.00	2.57	0.94	0.59	0
**182**	9	*TOP2A*	0.00	2.44	0.89	0.56	0
**183**	9	*CENPF*	0.00	2.23	0.89	0.58	0
**184**	9	*ASPM*	0.00	2.09	0.86	0.53	0
**185**	9	*TPX2*	0.00	2.02	0.88	0.57	0
**186**	9	*CENPE*	0.00	1.62	0.78	0.44	0
**187**	9	*DIAPH3*	0.00	1.56	0.88	0.55	0
**188**	9	*ANLN*	0.00	1.56	0.82	0.49	0
**189**	9	*PRC1*	0.00	1.39	0.73	0.44	0
**190**	9	*KIF11*	0.00	1.37	0.77	0.49	0
**191**	9	*NUSAP1*	0.00	1.36	0.71	0.41	0
**192**	9	*CIT*	0.00	1.34	0.75	0.5	0
**193**	9	*KPNA2*	0.00	1.31	0.73	0.46	3.90947789658089E-290
**194**	9	*CDK1*	0.00	1.27	0.68	0.51	1.05324536288692E-221
**195**	9	*KIF4A*	0.00	1.26	0.71	0.49	0
**196**	9	*NCAPG*	0.00	1.19	0.7	0.5	6.08843872357421E-298
**197**	9	*CEP128*	0.00	1.18	0.79	0.55	3.67991367428791E-298
**198**	9	*KIF14*	0.00	1.17	0.66	0.45	1.75116932862194E-259
**199**	9	*CKAP2L*	0.00	1.16	0.69	0.51	7.57255848194669E-251
**200**	9	*MIR924HG*	0.00	1.10	0.72	0.57	6.10640620425961E-203
**201**	10	*MMP9*	0.00	2.77	0.67	0.53	8.08397127732943E-193
**202**	10	*LAMC2*	0.00	2.72	0.97	0.74	0
**203**	10	*KRT6A*	0.00	2.63	0.89	0.67	0
**204**	10	*LAMA3*	0.00	2.51	0.8	0.65	2.88170788687901E-275
**205**	10	*LAMB3*	0.00	1.81	0.8	0.62	3.27852024209837E-276
**206**	10	*KRT17*	0.00	1.79	0.49	0.51	1.15843945877946E-40
**207**	10	*COL17A1*	0.00	1.77	0.68	0.59	5.38500051358062E-140
**208**	10	*INPP4B*	0.00	1.35	0.81	0.63	2.04825297095848E-212
**209**	10	*KRT14*	0.00	1.22	0.51	0.59	3.23324533571871E-21
**210**	10	*TMEM132D*	0.00	1.22	0.62	0.56	1.35736286299835E-92
**211**	10	*SEMA3C*	0.00	1.15	0.95	0.8	1.78100630981075E-230

**212**	10	*THBS1*	0.00	1.13	0.88	0.7	1.06459363129595E-135
**213**	10	*DSC2*	0.00	1.13	0.85	0.68	9.22506907450692E-173
**214**	10	*ITGB4*	0.00	1.12	0.49	0.45	3.83789725369348E-66
**215**	10	*TNS4*	0.00	1.09	0.42	0.52	1.58597324817715E-08
**216**	10	*NTN4*	0.00	1.09	0.69	0.56	8.74503754757718E-152
**217**	10	*ITGA6*	0.00	1.08	0.66	0.63	4.50495301171499E-66
**218**	10	*LINC00511*	0.00	1.08	0.84	0.57	1.39860819563529E-216
**219**	10	*FRMD6*	0.00	1.06	0.78	0.61	8.84377502264119E-144
**220**	10	*CHST11*	0.00	1.05	0.83	0.67	5.58462409190851E-165
